# Interaction between CD36 and FABP4 modulates adipocyte-induced fatty acid import and metabolism in breast cancer

**DOI:** 10.1038/s41523-021-00324-7

**Published:** 2021-09-24

**Authors:** Jones Gyamfi, Joo Hye Yeo, Doru Kwon, Byung Soh Min, Yoon Jin Cha, Ja Seung Koo, Joon Jeong, Jinu Lee, Junjeong Choi

**Affiliations:** 1 College of Pharmacy, Yonsei Institute of Pharmaceutical Sciences, Yonsei University, Incheon, Korea; 2 Department of Medical Laboratory Science, University of Health and Allied Sciences, Ho, Ghana; 3 Department of Surgery, Yonsei University College of Medicine, Seoul, Republic of Korea; 4 Department of Pathology, Gangnam Severance Hospital, Yonsei University College of Medicine, Seoul, Korea; 5 Department of Pathology, Yonsei University College of Medicine, Seoul, Republic of Korea; 6 Department of Surgery, Gangnam Severance Hospital, Yonsei University College of Medicine, Seoul, Korea

**Keywords:** Breast cancer, Breast cancer, Prognostic markers

## Abstract

Adipocytes influence breast cancer behaviour via fatty acid release into the tumour microenvironment. Co-culturing human adipocytes and breast cancer cells increased CD36 expression, with fatty acid import into breast cancer cells. Genetic ablation of CD36 attenuates adipocyte-induced epithelial-mesenchymal transition (EMT) and stemness. We show a feedforward loop between CD36 and STAT3; where CD36 activates STAT3 signalling and STAT3 binds to the CD36 promoter, regulating its expression. CD36 expression results in metabolic reprogramming, with a shift towards fatty acid oxidation. CD36 inhibition induces de novo lipogenesis in breast cancer cells. Increased CD36 expression occurs with increased FABP4 expression. We showed that CD36 directly interacts with FABP4 to regulate fatty acid import, transport, and metabolism. CD36 and FABP4 inhibition induces apoptosis in tumour cells. These results indicate that CD36 mediates fatty acid import from adipocytes into cancer cells and activates signalling pathways that drive tumour progression. Targeting CD36 may have a potential for therapy, which will target the tumour microenvironment.

## Introduction

The tumour microenvironment is key to tumour growth. In breast cancer, the role of adipocytes has gained enormous attention due to their proximity to the developing breast tumour. Clinical studies have associated breast cancer invasion of surrounding adipocytes at the tumour margin, with poor patient outcomes^1,2^. Focus on elucidating the mechanism by which adipocyte influence cancer cell growth has identified two major mechanisms. Firstly, adipocytes secrete adipocytokines (adipocyte-secreted growth factors and cytokines), which activate growth-promoting signalling pathways in tumour cells and drive various oncogenic processes. Adipocyte-secreted interleukin-6 (IL-6) and leptin reportedly induce and regulate epithelial–mesenchymal transition (EMT) in cancer cells^3–5^. Adipocyte-secreted leptin activates targets that enhance stem cell renewal and chemoresistance^6,7^. Secondly, adipocytes release metabolites and biomolecules, which remodel tumour cell metabolism to enhance tumour growth. Energy metabolism reprogramming is a key hallmark of tumour growth^8,9^. Adipocytes proximity to breast cancer cells allows breast cancer cells to parasite on their energy substrates and obtain metabolites, including lactate, glutamine and fatty acids, which are limited in the growing tumour environment^9–11^. The acquired metabolites maintain the metabolic needs and provide a selective advantage for tumour survival and progression in the harsh microenvironment. There are emerging studies into the mechanisms of free fatty acid (FFA) import and metabolism in breast cancer microenvironment, however, several aspects of the process remain unexplored.

Fatty acid translocase/CD36 is a key membrane glycoprotein involved in importing adipocyte-released fatty acid into breast cancer cells^12,13^. CD36 is expressed in a wide range of cells, including platelets, myocytes, monocytes, macrophages, adipocytes and some epithelial cells^12,13^. The primary functions of CD36 include fatty acid uptake, cell adhesion or a class B scavenger receptor^12,13^. Recently, the functions of CD36 have extended to include lipid metabolism, inflammatory response, and cancer development^12^. Studies across various cancers (gastric, oral squamous cell, prostate and ovarian) have highlighted the role of CD36 in fatty acid import into cancer cells and fatty acid oxidation (FAO) regulation to enhance progression^14–16^. The myriad of roles linked to CD36 indicate a context-dependent function. In adipocyte-breast cancer cell interaction, CD36 plays a role in reprogramming metabolism, with a shift towards enhanced FAO^11,13^. Thus, CD36 may be a unique receptor for fatty acid import and regulate various oncogenic processes. However, the molecular mechanisms and regulators of CD36 activity in breast cancer cells remain largely unexplored. This study aimed to explore the context-dependent role of CD36 in adipocyte-breast cancer interaction. To this, we report an increased CD36 expression in breast cancer tissues invading surrounding adipose tissues and its tumour promoting function in adipocyte-breast cancer cell interaction.

## Results

### CD36 is key in adipocyte-released fatty acid import into breast cancer cells

**Fig. 1:**
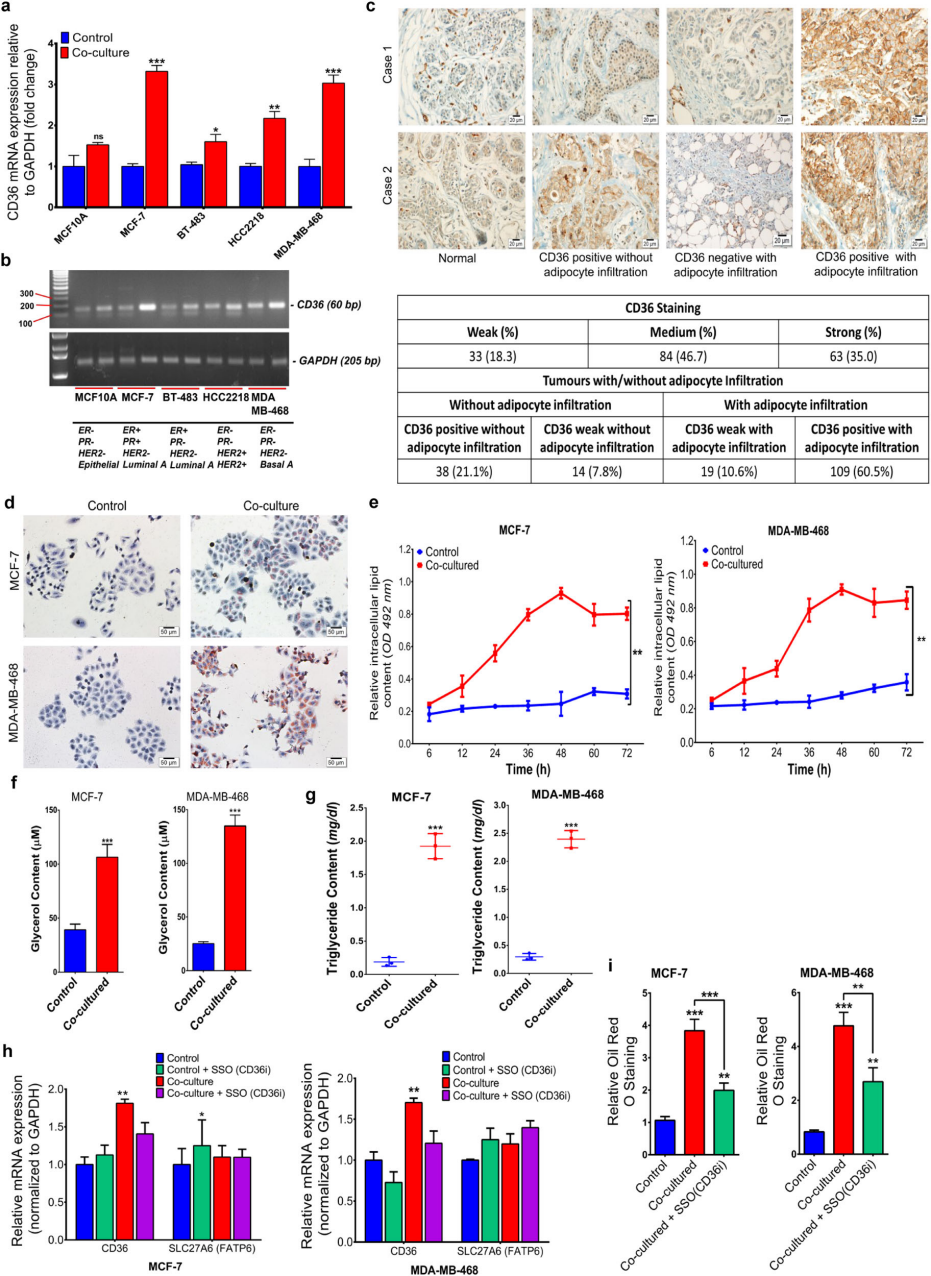
CD36 is key in adipocyte-released fatty acid import into breast cancer cells. **A**, **B** Expression of CD36 was examined by qRT-PCR (a) and western blot (b) in breast cancer cell lines with different molecular characteristics. **C** Representative IHC images for CD36 expression in breast tissues with/without adipocyte infiltration, showing percentage of tissues with weak, medium and strong CD36 staining/adipocyte infiltration. **D** Representative images of Oil Red O stained MCF-7 and MDA-MB-468 cells following co-culture with differentiated human adipocytes. **E** Quantitative estimation of the rate of fatty acid accumulation in MCF-7 and MDA-MB-468 breast cancer cells at specific time points (0, 6, 12, 24, 36, 48, 60 and 72 h) following co-culture. **F** Quantification of intracellular glycerol content in MCF-7 and MDA-MB-468 cultured with/without adipocytes for 48 h. **G** Quantification of intracellular triglycerol content in MCF-7 and MDA-MB-468 cultured with/without adipocytes for 48 h. **H** Comparison between CD36 and SLC27A6 mRNA expression in MCF-7 and MDA-MB-468 cells cultured with/without adipocyte CM treated with/without the CD36 inhibitor; Sulfosuccinimidyl oleate (SSO). **I** Quantitative estimation of accumulated fatty acid following Oil Red O staining in MCF-7 and MDA-MB-468 co-cultured with/without SSO. Relative mRNA expression was normalised to GAPDH experimental results are representative of three independent experiments. (qRT-PCR data represent mean ± SEM., ***p < 0.001; **p < 0.01; *p < 0.05, *n* = 3, quantification data indicate mean ± SD; ***p < 0,001; **p < 0.01; *p < 0.05).

To explore the context-dependent role of CD36 in adipocyte-breast cancer cell interaction, CD36 expression was examined in a direct co-culture of breast cancer cells (MCF10A, MCF-7, BT-483, HCC2218 and MDA-MB-468) and differentiated human adipocytes (hADS) after 48 hours via qRT-PCR. CD36 expression increased in the co-cultured cells, compared to the control groups (Fig. 1A). The increased CD36 expression was higher in the luminal MCF-7 and basal MDA-MB-468 cell lines, compared to the luminal BT-483 and HER2+ HCC2218 cell lines (Fig. 1A, B). In human tissue microarray, consisting of normal and 180 human breast tumour samples, immunohistochemistry (IHC) revealed weak or no CD36 expression in seven of the nine normal patient tissues. CD36 expression varied in tumour tissues, with 33 (18.3%) having weak or no CD36 expression. Moderate CD36 staining was observed in 84 (46.7%) of tumour tissues and strong staining was observed in 63 (35%) of tumour tissues (Fig. 1C). Adipocyte infiltration by tumour cells was present in 128 (71.1%) of tumour tissues, positive CD36 expression was present in 109 (60.5%) of tumour tissues (Fig. 1C) with adipocyte infiltration. Weak or no CD36 expression was present 19 (10.6) of tumour tissues with adipocyte infiltration (Fig. 1C).

The direct role of CD36 in fatty acid import was investigated by indirect co-culture of MCF-7 and MDA-MB-468 cells with adipocyte-conditioned media, with/without 200 μM of the CD36 inhibitor Sulfosuccinimidyl oleate sodium (SSO). Oil red O staining of co-cultured cells revealed phenotypic changes, with tiny intracellular lipid droplet formation (Fig. 1D). Lipid accumulation in cells increased from 12 h, after co-culture, and peaked between 36 and 48 h (Fig. 1E, F). Expectedly, intracellular glycerol and triglyceride contents significantly increased in co-cultured cells (Fig. 1G and Supplementary Fig. 1), while CD36 inhibition decreased fatty acid accumulation (Fig. 1H, I and Supplementary Fig. 1H–L). These results indicate the active role of CD36 in fatty acid import in adipocyte-breast cancer cell interaction.

### CD36 mediates adipocyte-induced EMT emergence and stem cell traits

To examine the molecular function of CD36 in adipocyte-breast cancer cell interaction, we established stable CD36 expression MCF-7 and MDA-MB-468 cell lines (CD36 expression cells). Stable CD36-knockout MCF-7 and MDA-MB-468 cells, using two distinct CRISPR-Cas gRNA targeting CD36 (CD36-knockout1 and CD36 knockout2). Control MCF-7 and MDA-MB-468 cell lines transfected, with an empty pLVX-EIP plasmid (mock-CD36). The efficiency of CD36 knockout and amplification were validated by western blot and qRT-PCR (Supplementary Fig. 2).

**Fig. 2:**
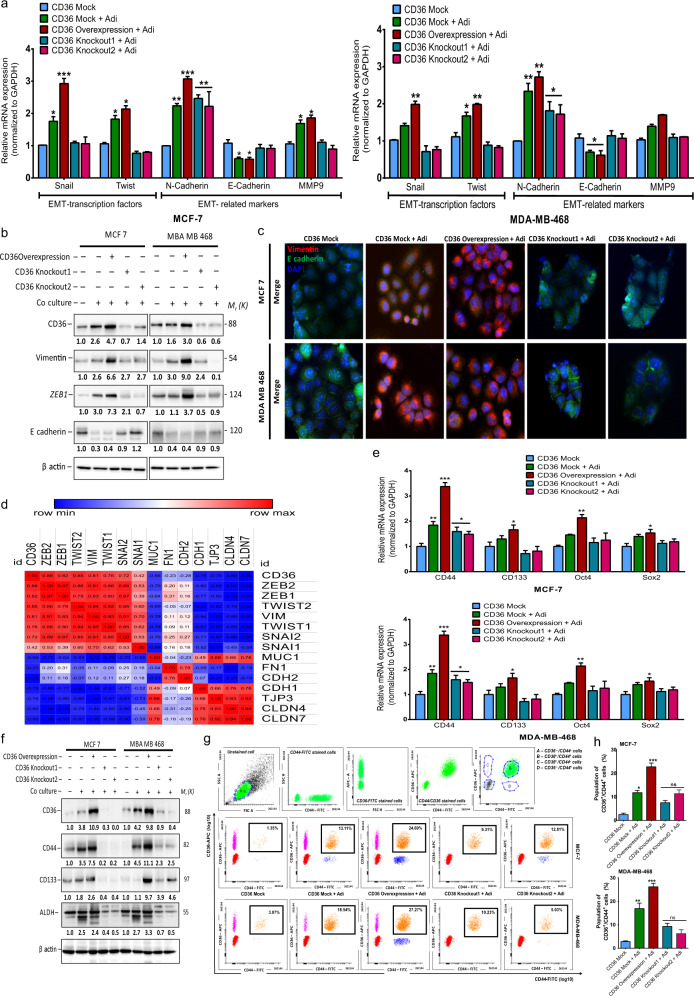
Genetic ablation of CD36 attenuates adipocyte-induced EMT and stemness in breast cancer cells. **A** Quantitative real-time-PCR (qRT-PCR) comparing the expression of EMT-TFs (TWIST1 and SNAIL) and EMT-related genes (MMP9, N-cadherin and E-cadherin) in MCF-7 and MDA-MB-468 cells co-cultured with adipocytes. **B** Expression of CD36 and EMT related markers (vimentin, ZEB1 and e-cadherin) in cells following CD36 knockout and co-culture with adipocytes was analysed by western blotting. **C** Representative immunofluorescence images of vimentin (red) and e-cadherin (green) in cells. Nucleus was stained with DAPI. **D** Correlation matrix showing the association between CD36 and EMT-related markers. **E** qRT-PCR comparing the expression of stem cell markers (CD44, CD133, Oct4 and Sox2) in MCF-7 and MDA-MB-468 cells co-cultured with adipocytes. **F** Expression of CD36 and stem cell markers (CD44, CD133 and ALDH) in cells following CD36 knockout and co-culture with adipocytes was analysed by western blotting. **G** MCF-7 and MDA-MB-468 cell populations was sorted by flow cytometry for CD36^+^/CD44^+^ cells after co-culture with adipocytes. **H** Percentage of cells with positive CD36/CD44 expression in MCF-7 and MDA-MB-468 after co-culture with adipocytes. (qRT-PCR data represent mean ± SEM., ***p < 0,001; **p < 0.01; *p < 0.05, *n* = 3. Quantitative data indicate mean ± SD; ***p < 0,001; **p < 0.01; *p < 0.05) (**I**, **J**).

Elevated CD36 expression is linked to fatty acid uptake and EMT emergence^15^. We examined the association between increased CD36 expression and the expression of EMT transcription factors (EMT-TFs: TWIST1 and SNAIL) and EMT-related markers (EMT-RMs: N-cadherin, E-cadherin and MMP9) in co-cultured MCF-7 and MDA-MB-468 cells. Co-cultured CD36-expressing cells had upregulated expression of the EMT markers Twist, Snail, and N-cadherin compared, to the mock-CD36 cells (Fig. 2A). Western blot analysis of EMT markers showed upregulated protein expression of vimentin and ZEB1, with decreased E-cadherin expression, in co-cultured cells (Fig. 2B). Immunofluorescence images showed increased vimentin staining and decreased E-cadherin staining in CD36 expression and co-cultured cells (Fig. 2C). We used the TCGA breast cancer cohort to interrogate the correlation between increased CD36 expression and EMT markers in clinical breast cancer patients. We obtained EMT scores, as described by Nath et al.^15^ (Supplementary Fig. 2A, B). The correlation matrix for CD36 and EMT markers revealed a strong positive correlation between increased CD36 expression and mesenchymal markers expression (Fig. 2D and Supplementary Fig. 3A, B) and a strong negative correlation between increased CD36 expression and epithelial markers expression (Fig. 2D and Supplementary Fig. 3A, B).

Since EMT emergence is strongly linked to stem-cell trait development^17^, these findings indicate the potential role of CD36 in stemness; hence, we investigated the influence of CD36 expression on the emergence of stem cell traits, following direct co-culturing. Co-cultured mock-CD36 and CD36 expression MCF-7 and MDA-MB-468 cells had significantly increased mRNA levels of the stem cell markers CD44 and Oct4 (Fig. 2E). CD36 knockouts significantly decreased the expression of stem-cell markers (Fig. 2E). Co-cultured cells also showed upregulated protein levels of the stemness markers CD44, CD133 and ALDH (Fig. 2F). Co-cultured mock-CD36, CD36 expression and CD36-knockout cells were sorted, using fluorescence-activated cell sorting (FACS), to determine CD36-positive (CD36^+^) only, CD44-positive (CD44^+^) only and CD36/CD44-positive (CD36^+^/CD44^+^) cell populations. Expression of stem-cell-like traits occurred only in a subpopulation of co-cultured cells. In mock-CD36 cells, the CD36^+^/CD44^+^ cell population was significantly fewer (Fig. 2G, H), compared to the co-cultured mock-CD36 and CD36 expression cells. The CD36^+^/CD44^+^ cell population increased significantly (Fig. 2G, H). However, the CD36^+^/CD44^+^ cell population in the co-cultured knockout-CD36 cells significantly decreased (Fig. 2G, H). These results indicate that the emergence of stem-cell-like traits in breast cancer cells is influenced in part by the adipocytes, and upregulated CD36 expression enhances the emergence of the CD36^+^/CD44^+^ cell population. Together, these results indicate a role of CD36 in adipocyte-induced EMT and stemness. Genetic ablation of CD36 inhibits expression of EMT and stemness markers.

### Genetic ablation of CD36 inhibits adipocyte-induced tumour growth and tumoursphere formation

**Fig. 3:**
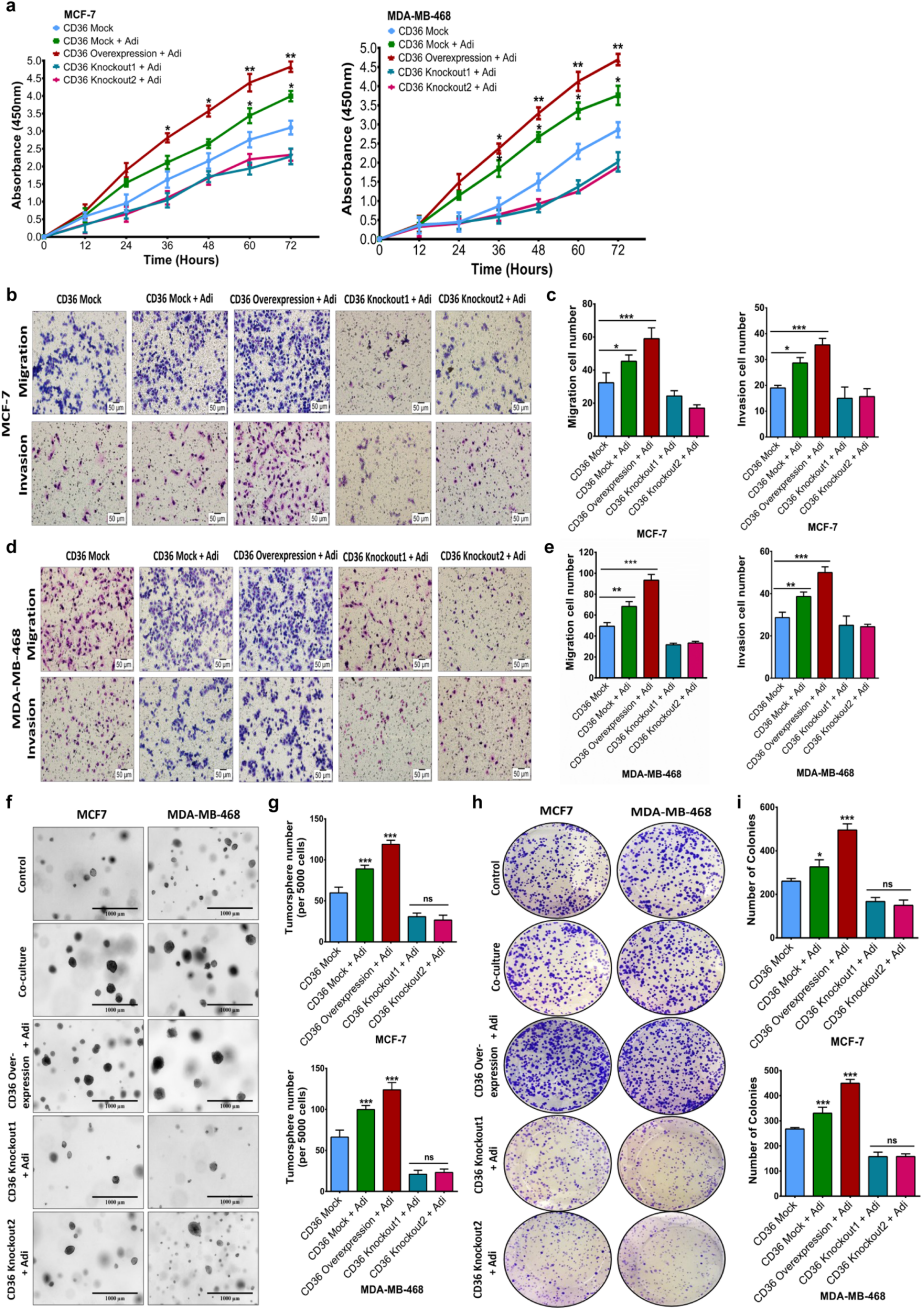
Genetic ablation of CD36 inhibits adipocyte-induced tumour growth and tumoursphere formation. **A** Evaluation of effect of CD36 ablation on proliferation of MCF-7 and MDA-MB-468 breast cancer cells co-cultured with adipocytes for 48 h. Representative images of MCF-7 (**B**) and MDA-MB-468 (**D**) migration and invasion (×200 magnification) after co-culture with differentiated human adipocytes. Quantitative analysis of migration and invasion cell numbers of MCF-7 (**C**) and MDA-MB-468 (**E**) breast cancer cells following CD36 ablation and co-culture with adipocytes. **F**, **G** Evaluation of the tumour formation capabilities of CD36 expression MCF-7 and MDA-MB-468 cells co-cultured with/without adipocytes. Representative images of spheroid formation ability of MCF-7 and MDA-MB-468 cells (**F**). Quantitative analysis of number of spheroids formed by MCF-7 and MDA-MB-468 cells (**G**). **H** Representative images of colony formation ability of CD36 expression MCF-7 and MDA-MB-468 breast cancer cells after co-culture with/without adipocytes. **I** Quantitative analysis of the number of colonies formed by MCF-7 and MDA-MB-468 cells. All results are representative of three independent experiments. (Data indicate mean ± SD; ***p < 0.001; **p < 0.01; *p < 0.05).

The role of CD36 in promoting adipocyte-induced EMT and stemness indicates a direct role in adipocyte-induced breast cancer cells aggressiveness. Hence, we determined if the genetic ablation of CD36 limits adipocyte-induced proliferation, migration, invasion, and tumorigenesis. Co-cultured CD36-knockout cells had significantly decreased proliferative, migratory, and invasive characteristics (Fig. 3A–E). We also examined the effect of CD36 on breast cancer cell motility. Co-cultured CD36-knockout cells had significantly decreased motility (Supplementary Fig. 3C, D). Co-cultured mock-CD36 and CD36 expression cells formed more spheroids, compared with the control group (Fig. 3F, G). CD36-knockout cells formed inconspicuous and fewer spheroids (Fig. 3F). Similar findings were observed in the mock-CD36 and CD36-knockout cells via anchorage formation assay (Fig. 3H, I). Collectively, these results indicate that CD36 upregulation enhances the proliferative, migratory, and invasive capabilities of breast cancer in adipocyte-breast cancer cell interaction.

### CD36 induces stemness via activation of the STAT3 signalling axis

**Fig. 4:**
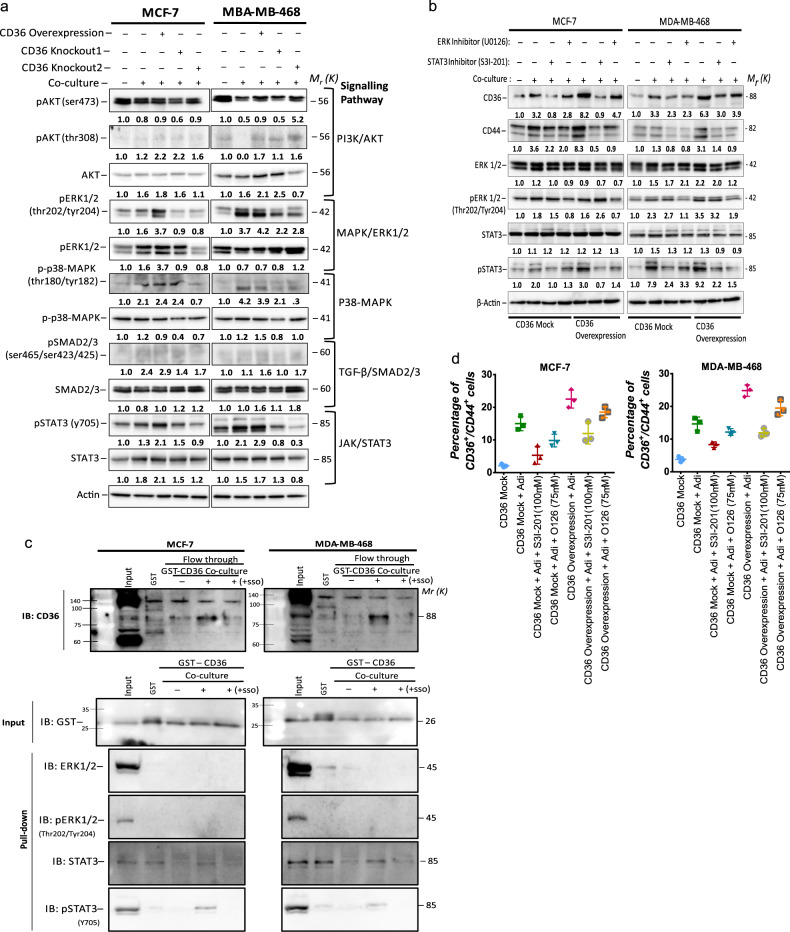
Upregulation of CD36 occurs with activation of the ERK1/2 and STAT3 signalling pathway. **A** Expression of key signalling pathway targets (PI3K/AKT, TGF-β/SMAD, MAPK/ERK1/2, p38 MAPK and JAK/STAT3) involved in tumour progression in cells following overexpression and genetic ablation of CD36 was analysed by western blotting. **B** Expression of CD36, CD44, ERK1/2, pERK1/2, STAT3 and pSTAT3 in MCF-7 and MDA-MB-468 cell following treatment with the ERK1/2 inhibitor (UO126) and STAT3 inhibitors (S3I-201), analysed by western blotting. **C** Representative western bolt of GST pull-down assay indicating CD36 directly interacts with STAT3. **D** Percentage of cells with CD36^+^/CD44^+^ expression in MCF-7 and MDA-MB-468 after co-culture with adipocytes and treated with/without U0126 and S3I-201. Experimental results are representative of three independent experiments. (Data indicate mean ± SD; ***p < 0,001; **p < 0.01; *p < 0.05).

To identify signalling pathways activated, following co-culturing, and potentially involved in CD36-dependent induction of EMT and stemness, we determined the levels of various oncogenic pathway proteins in adipocyte co-cultured breast cancer cells, with abrogated CD36 activity, by western blotting (Fig. 4A). The PI3K/AKT, ERK1/2 and STAT3 signalling pathways were activated in MCF-7 cells and PI3K/AKT, ERK1/2, p38-MAPK and STAT3 signalling pathways in MDA-MB-468 cells, with increased expression of phosphorylated forms (Fig. 4A). To identify pathways directly linked to CD36 activity, we focused on pathways activated in co-cultured CD36 expression cells and repressed in CD36-knockout cells. The ERK1/2 and STAT3 pathways were the only pathways increased in the CD36-expressing cells and decreased, with repressed CD36 activity (Fig. 4A).

To validate the direct involvement of the identified pathways in CD36-dependent effects, we inhibited ERK1/2 and STAT3 using the selective STAT3 (S3I-201) and ERK1/2 (U0126) inhibitors and determined their effect on CD36 expression and/or activity following indirect co-culture. CD36, CD44, pSTAT3 and pERK1/2 protein levels increased in cells cultured in adipocyte-conditioned media, without S3I-201 or U0126, and inhibiting the pathways decreased CD36 and CD44 expressions (Fig. 4B). However, inhibiting STAT3 activity had a more significant decrease on CD36 protein levels, compared to ERK1/2 inhibition (Fig. 4B and Supplementary Fig. 4A–D). Cell signalling activity of CD36 is speculated to be mediated by the Tyr-468 and Cys-464 residues at the C-terminus, which interact with Src-protein tyrosine kinases^13^. Hence, we determined if CD36-induced STAT3 or ERK1/2 activity involves direct interaction of CD36 with the signalling molecules. GST-pull-down assay for STAT3, pSTAT3 (Y705), ERK1/2 and pERK1/2 (Thr202/Tyr204) showed a direct interaction between CD36, STAT3 and pSTAT3 in both MCF-7 and MDA-MB-468 cells (Fig. 4C). Expectedly, CD36 did not show any direct interaction with ERK1/2 and pERK1/2 since their activation usually involves intermediates that do not directly interact with transmembrane receptors, unlike STAT3, which directly interacts with transmembrane receptors and translocate to the nucleus once phosphorylated (Fig. 4C).

We further evaluated the potential role of the STAT3 and ERK1/2 signalling axis in the emergence of CD36/CD44^+^ cells. We cultured mock-CD36 and CD36 expression cells, with adipocyte-conditioned media and S3I-201 or U0126, for 48 h, subjected them to flow cytometry, and the CD36/CD44^+^ cell population determined. S3I-201 treatment decreased the CD36/CD44^+^ cell population in the mock-CD36 and CD36 expression cells (Fig. 4D). Both STAT3 and ERK1/2 inhibition decreased the CD36/CD44^+^ cell population in the mock-CD36 and CD36 expression cells (Fig. 4D). However, STAT3 inhibition produced a greater decrease in CD36 protein expression and fewer CD36/CD44^+^ cell population compared to ERK1/2 inhibition. Altogether, these results indicate that STAT3 and ERK1/2 inhibition decrease CD36 expression in co-cultured breast cancer cells. STAT3 inhibition have a greater effect on CD36 expression and the emergence of stem cell traits.

### Phosphorylated STAT3 binds to the CD36 promoter and regulates CD36 expression

**Fig. 5:**
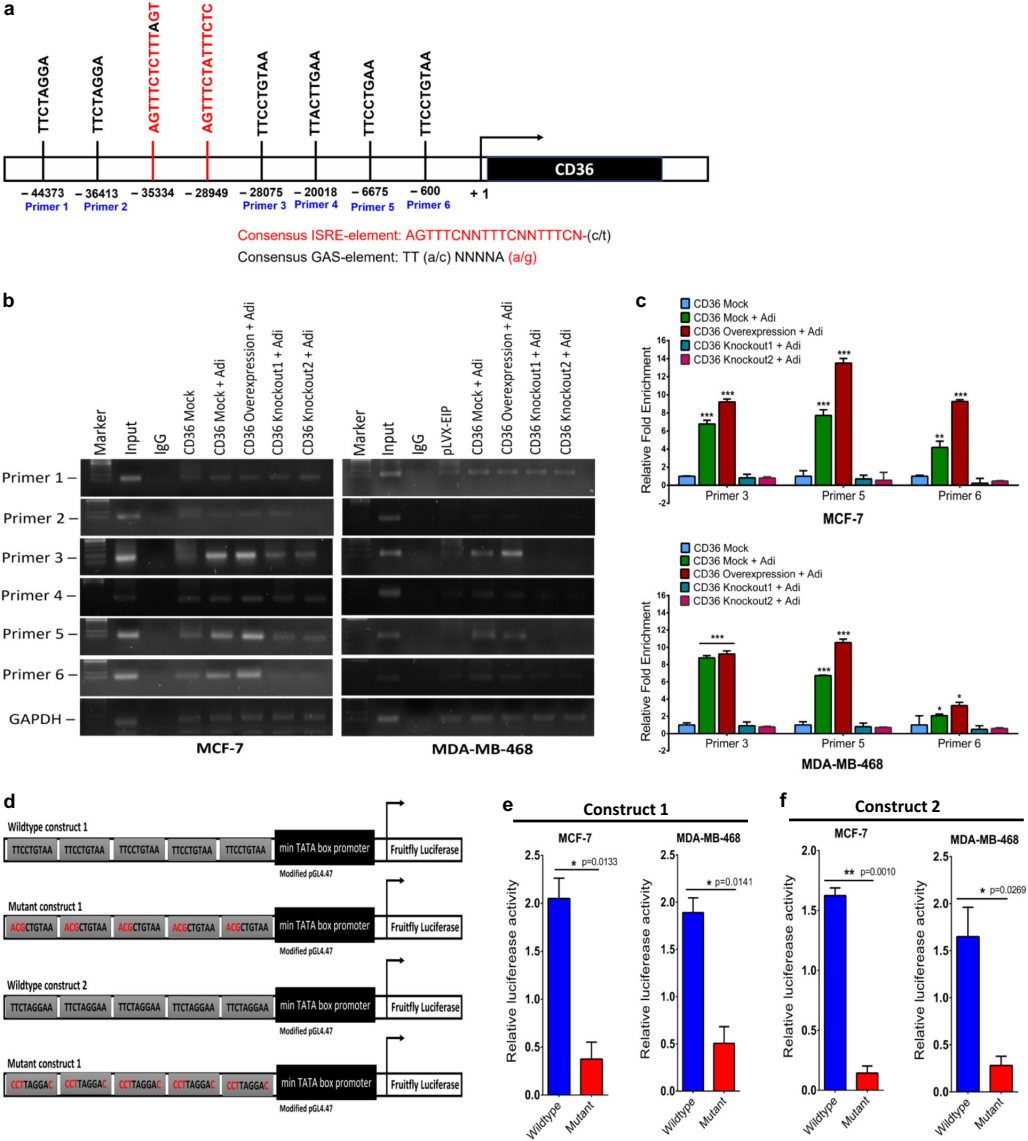
STAT3 binds to the CD36 promoter and regulate CD36 expression. **A** Schematic representation of the location of identified STAT3 GAS- and ISRE-elements in the CD36 promoter. **B** Chromatin immunoprecipitation (ChIP) assay on the promoter of CD36, showing pSTAT3 bound to predicted CD36-binding sites. GAPDH is shown as a negative control. **C** qRT-PCR of ChIP assay in MCF-7 and MDA-MB-468 cells. CD36 promoter occupancy by STAT3 primers 3, 5 and 6 is increased with adipocytes co-culture but is strongly reduced in CD36 ablated cells. **D** A schematic diagram of the STAT3 consensus sequence luciferase constructs 1 and 2 and mutant constructs 1 and 2. **E**, **F** Luciferase constructs were stimulated with IL-6 (50 ng) to induce luciferase activity. Relative luciferase activity was normalised against a non-inducible luciferase construct. All results are representative of three independent experiments. Relative mRNA expression was normalised to GAPDH experimental results are representative of three independent experiments. (qRT-PCR data indicate mean ± SEM; ***p < 0,001; **p < 0.01; *p < 0.05. Relative luciferase activity data indicate mean ± SD; ***p < 0,001; **p < 0.01; *p < 0.05).

Since STAT3 inhibition decreases CD36 protein expression, we determined if STAT3 regulates CD36 transcriptional expression. STAT3 phosphorylation and activation allow binding to the STAT consensus binding sequences (GAS motif), with the sequence TT-(A/C)-NNNNA-(A/G) and activate the transcription of their target genes. We located six unique STAT3 consensus sequences within the CD36 promoter before the putative start site (Fig. 5A). The CD36 promoter also contains two distinct interferon-sensitive response elements (AGTTTCNNTTTCN -(C/T)), which are binding sites for various STAT proteins involved in gene activation (Fig. 5A). To determine if the identified binding sites regulate CD36 expression, we designed Chip-primers spanning the six identified STAT3 consensus sequences (Supplementary Table 3) and performed a chip assay. Co-cultured mock-CD36 and CD36 expression MCF-7 and MDA-MB-468 cells had significant enrichment with primers 3 and 5, but marginal for primer 6. Enrichment for primers 3, 5 and 6 decreased in the CD36-knockout cells (Fig. 5B, C).

The presence of unique STAT3 GAS-element in the CD36 promoter indicates the potential for STAT3 to regulate CD36 expression. To test this, the SIE site in the pGL4.4 (luc2P/SIE/hygro) plasmid was replaced, with 5 repeating STAT3 promoter sequence #3 (wildtype -construct 1), #5 (wildtype construct 2) and mutant forms (Fig. 5D). We transfected MCF-7 and MDA-MB-468 cells, with the wildtype, and mutant STAT3 promoter constructs (1 and 2), stimulated them with 50 ng IL-6, and determined the luciferase activity of both constructs. Cells transfected, with wildtype constructs (1 and 2), had significantly increased luciferase activity after IL-6 stimulation, compared to cells transfected with the mutant constructs (1 and 2) (Fig. 5E, F). Thus, the identified STAT3 consensus sequences (3 and 5) in the CD36 promoter can stimulate luciferase gene expression. These results indicate that pSTAT3 induces CD36 transcription by binding to its putative consensus sequence in the CD36 promoter.

### CD36 regulates metabolic reprogramming towards fatty acid oxidation

**Fig. 6:**
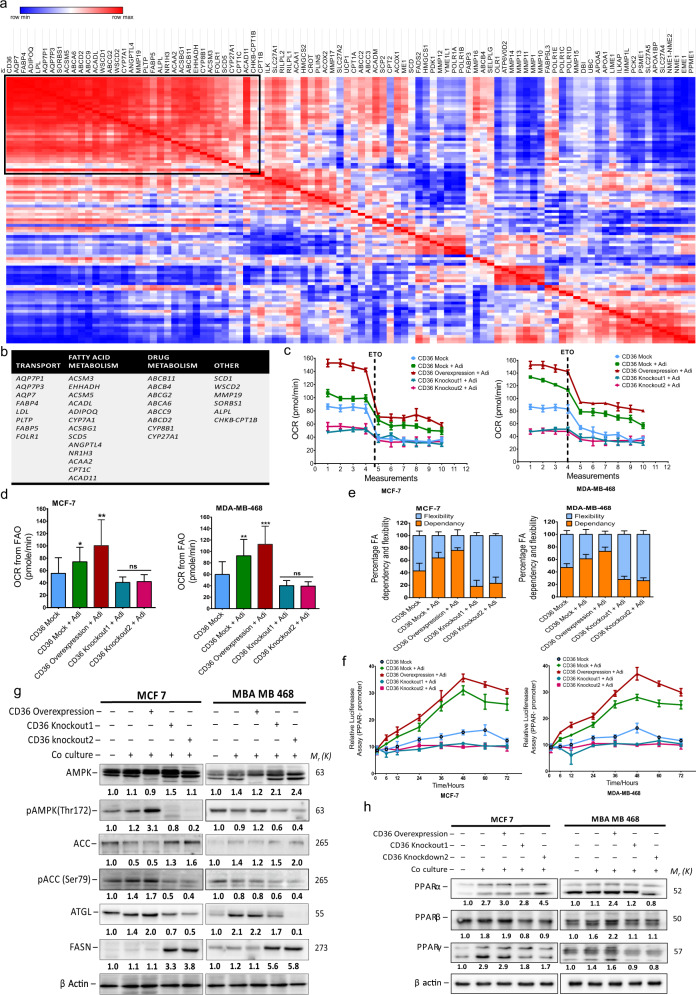
CD36 regulates metabolic reprogramming towards fatty acid oxidation. **A** Correlation matrix heatmap showing the association between CD36 mRNA expression *z*-scores (TCGA breast cancer dataset) with genes related to fatty acid metabolic activity. **B** Classification of positively correlated genes into groups based on their associated functions. **C** XFe24 Seahorse FAO assay in MCF-7 and MDA-MB-468 cells with/without CD36 ablation cultured with/without adipocytes in real time under basal conditions and in response to mitochondrial CPT1 inhibitor (Etomoxir). **D** The FAO OCR rate of MCF-7 and MDA-MB-468 cells with/without CD36 ablation cultured with/without adipocytes (Data indicate mean of three time points ± SD; *p* values were calculated using Student’s *t* test, ***p < 0,001; **p < 0.01; *p < 0.05). **E** OCR was measured during the Seahorse XF Mito-Fuel Flex assay. The percentage of dependence on fatty acids was calculated by quantifying the change in basal OCR after fatty acid oxidation was blocked using 4 μM etomoxir and divided by the total change in OCR from baseline after combined inhibition of fatty acid, glutamine and pyruvate oxidation using 4 μM etomoxir, 3 μM BPTES and 2 μM UK5099, respectively. Fatty acid fuel flexibility was calculated by measuring the change in sensitivity to etomoxir’s inhibition of OCR after blockade of glutamine and glucose oxidation and represents the ability of MCF-7 and MDA-MB-468 cells to increase oxidation of fatty acid when glutamine and pyruvate utilisation is precluded. **F** Relative luciferase activity in MCF-7 and MDA-MB-468 cells transfected with PPAR luciferase reporter plasmid and cultured with/without adipocytes. Luciferase activity was normalised against a non-inducible luciferase construct. **G** Expression of AMPK, pAMPK, ACC, pACC, ATGL and FASN in MCF-7 and MDA-MB-468 cells with/without CD36 genetic ablation and co-cultured with adipocytes analysed by western blotting. **H** Expression of PPARα, PPARβ and PPARγ in MCF-7 and MDA-MB-468 cells with/without CD36 genetic ablation and co-cultured with adipocytes analysed by western blotting. Relative mRNA expression was normalised to GAPDH. Experimental results are representative of three independent experiments. (Data indicate mean ± SEM.; ***p < 0,001; **p < 0.01; *p < 0.05).

To determine if increased CD36 expression results in metabolic changes, we used the GO-lipid metabolic process gene list present in the molecular signature database (MSigDB) to generate a correlation matrix for increased CD36 expression in the TCGA breast cancer patient dataset. We identified 26 key genes, with expression positively correlated, with increased CD36 expression (Fig. 6A and Supplementary Fig. 5A). Based on the ascribed gene function, the identified genes were put into four groups: Lipid and fatty acid transport (*AQP7P1, AQP7P3, AQP7, FABP4* and *LDL*), fatty acid metabolism (*ACSM3, EHHADH, ACSM5, ACADL, ADIPOQ, CYP7A1, ACSBG1* and *SCD5*), drug resistance (*ABCB11, ABCB4, ABCG2, ABCA6, ABCC9* and *ABCD2*) and others (*WSCD1, WSCD2, MMP19* and *SORBS1*) (Fig. 6B). Of note is the increased expression of AQP7 (aquaporin 7) and its isoforms (AQP7P1 and AQP7P3), which export glycerol from fatty acid metabolism; FABP4, which binds and shuttles fatty acids into cells and subcellular locations for metabolism; Acyl-CoA synthetases (*ACSM3, ACSM5)*, which activate fatty acids in the first step of their metabolism; and acyl-CoA dehydrogenase long-chain (*ACADL)*, which catalyses the first step of mitochondrial fatty acid beta-oxidation (Fig. 6B). The data suggest a causal relation between increased CD36 expression and cell energy reprogramming towards fatty acid metabolism, with increased expression of enzymes and transporters involved in FAO.

To validate the functional effect of CD36-induced fatty acid uptake on enhanced FAO and metabolic reprogramming in breast cancer cells, we assessed the metabolic changes following culturing with adipocyte-conditioned media, using the Seahorse XF24 analyser. We determined the energy phenotype of co-cultured mock-CD36, CD36 expression and CD36-knockout cells. We found that CD36 abrogation induced a quiescent energy state in the cells, with low glycolytic and metabolic respiratory pathways (Supplementary Fig. 5B). Co-cultured mock-CD36 and CD36-expressing cells assumed an energetic phenotype, indicating enhanced mitochondrial respiration (Supplementary Fig. 5B). Thus, breast cancer cells alter their metabolic programmes, with increased mitochondrial respiration, to meet their energy needs, with the available fatty acids. CD36 is key for this energy shift in breast cancer cells. CD36 knockout inhibits mitochondrial respiration and shifts the cell’s energy phenotype towards a quiescent state.

To investigate if the enhanced mitochondrial function in co-cultured cells is FAO-dependent, we measured the oxygen consumption rate (OCR) using the seahorse FAO assay kit. Basal OCR was significantly increased in co-cultured mock-CD36 and CD36-expressing cells (Fig. 5C, D). Treatment with carnitine-palmitoyltransferease-1 (CPT1) inhibitor, etomoxir (ETO) showed a sharp OCR decrease in the co-cultured mock-CD36 and CD36 expression cells (Fig. 5C, D and Supplementary Fig. 5E, F). These findings indicate enhanced FAO in co-cultured mock-CD36 and CD36 expression cells. We determined if the presence of excess FFA acids supplied by cancer-associated adipocytes induced a metabolic shift towards fatty acid fuel dependency using the seahorse mito-fuel flex assay. In mock-CD36 cells co-cultured with adipocytes, dependency on fatty acids doubled in comparison to control cells (Fig. 6E). While in CD36 knockouts dependency on fatty acid decreased to <26% (Fig. 6E). Co-cultured CD36 expression cells had over 70% dependency on fatty acids (Fig. 6E).

Mechanistically, overexpression of CD36 resulted in activation of the PPAR signalling axis, with increase PPARα and PPARγ expression (Fig. 6F, H). The shift towards fatty acid metabolism occurs with an increase protein expression of pAMPK, and ATGL, proteins that mediate fatty acid metabolism (Fig. 6G and Supplementary Fig. 6). While knockout of CD36 cells had increased protein expression of FASN, required for fatty acid synthesis (Fig. 6G and Supplementary Figs. 5 and 6). Taken together, these findings suggest a metabolic remodelling in breast cancer cells, in the presence of adipocytes. With increased expression of CD36 and proteins involved in fatty acid metabolism, which potentiates the shifts towards fatty acid metabolism, with enhanced mitochondrial respiration.

### CD36 directly interacts with FABP4 to mediate fatty acid localisation and metabolism

**Fig. 7:**
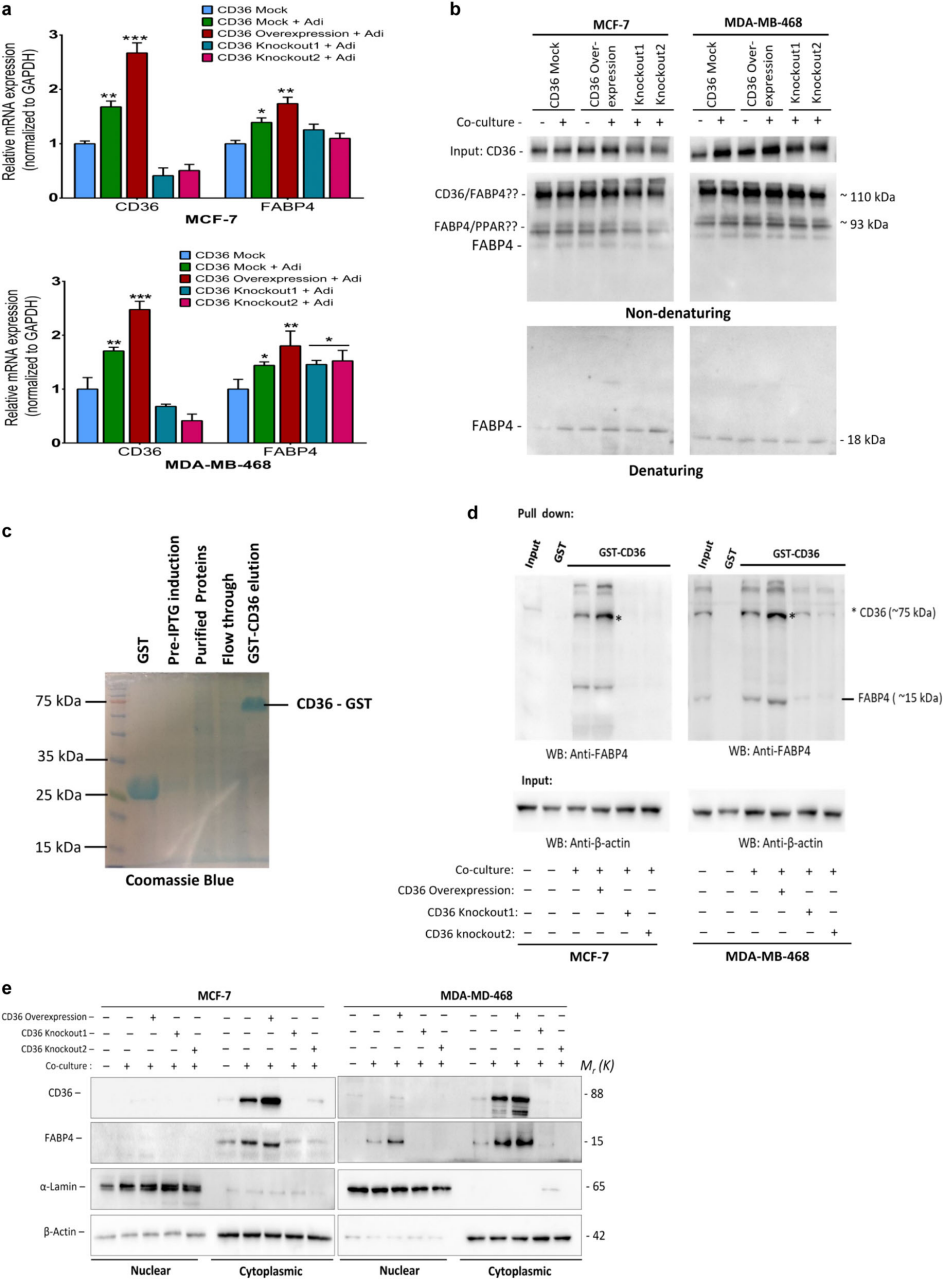
CD36 directly interacts with FABP4 to mediate fatty acid metabolism. **A** Quantitative real-time-PCR (qRT-PCR) comparing the expression of CD36 and FABP4 in MCF-7 and MDA-MB-468 co-cultured with adipocytes. **B** Immunoprecipitation with CD36 antibody revealed that FABP4 binds to CD36 in MCF-7 and MDA-MB-468 cell lines. **C** GST-tagged CD36 was immobilised to agarose beads after mixing with breast cancer cell lysate, eluted proteins were blotted with FABP4 antibody. Validation of GST-CD36 tagged protein by Coomassie blue staining. **D** Representative western blot of FABP4. FABP4 directly interacts with CD36. **E** Total nuclear and cytoplasmic levels of CD36 and FABP4 in MCF-7 and MDA-MB-468 cells cultured with/without adipocytes was analysed by western blot, α-lamin and β-actin were used as loading controls for nuclear and cytoplasmic fraction respectively. Relative mRNA expression was normalised to GAPDH. Experimental results are representative of three independent experiments. (qRT-PCR data are mean ± SEM, ***p < 0,001; **p < 0.01; *p < 0.05).

Based on our earlier results indicating a close association between CD36 and FABP4 (Fig. 6A), we compared their expression in the mock-CD36, CD36 expression and CD36-knockout cells, with/without adipocytes by qRT-PCR. Interestingly, both CD36 and FABP4 expressions increased in co-cultured cells (Fig. 7A). Next, we determined the correlation between increased CD36 expression and the various FABP genes (*FABP1*, *FABP2*, *FABP3*, *FABP4*, *FABP5* and *FABP6*) in the TCGA breast cancer cohort. A strong positive correlation existed between CD36 and FABP4, compared to other FABP forms (Supplementary Fig. 7B). To further explore this association, we combined the GO-fatty acid transmembrane transport, activity and binding activity dataset and generated a large dataset named the CD36-fatty acid partner dataset (Supplementary Fig. 7C). Using the TCGA database, we screened 16 tumour cohorts for the top 10 genes that positively correlated with increased CD36 expression. Interestingly, in the clinical datasets explored, FABP4 was the most common protein correlating with increased CD36 expression in 15 out of the 16 tumour types assessed (Supplementary Fig. 7C, D).

We hypothesised that the significant correlation between CD36 and FABP4 expression depended on their direct interaction to regulate fatty acid import and metabolism. To interrogate this, we performed an amino acid sequence alignment for CD36 and FABP4 and observed ten highly similar amino acid sequences and possible interacting motifs (Supplementary Fig. 7A). Non-denaturing immunoprecipitation assay revealed three distinct bands, indicating a possible interaction between CD36 and FABP4 and other proteins (Fig. 7B). Denaturing immunoprecipitation showed that CD36 specifically immunoprecipitated FABP4 (Fig. 7B). To validate this observation, we cloned the CD36 gene into the pGEX-4T-3 plasmid, followed by a pull-down assay, and blotting with an anti-FABP4 antibody. The results showed increased FABP4 pull-down in co-cultured CD36-expressing cells (Fig. 7C). We next examined the subcellular localisation of CD36 and FABP4. The cytoplasmic fraction had significantly increased CD36 and FABP4 protein levels in the co-cultured mock-CD36 and CD36-expression cells (Fig. 7D). Taken together, these results indicate a direct interaction between CD36 and FABP4 in the cytoplasmic region may regulate fatty acid import and metabolism. We can speculate from these findings that CD36 act as the transmembrane fatty acid importer, specifically involved in transmembrane fatty acid import FABP4 binds to fatty acids and interact with CD36 to import and shuttle them to various subcellular locations.

### Chemical inhibition of CD36 and FABP4 induces apoptosis in breast cancer cells and reduces primary tumour growth in xenograft mouse model

**Fig. 8:**
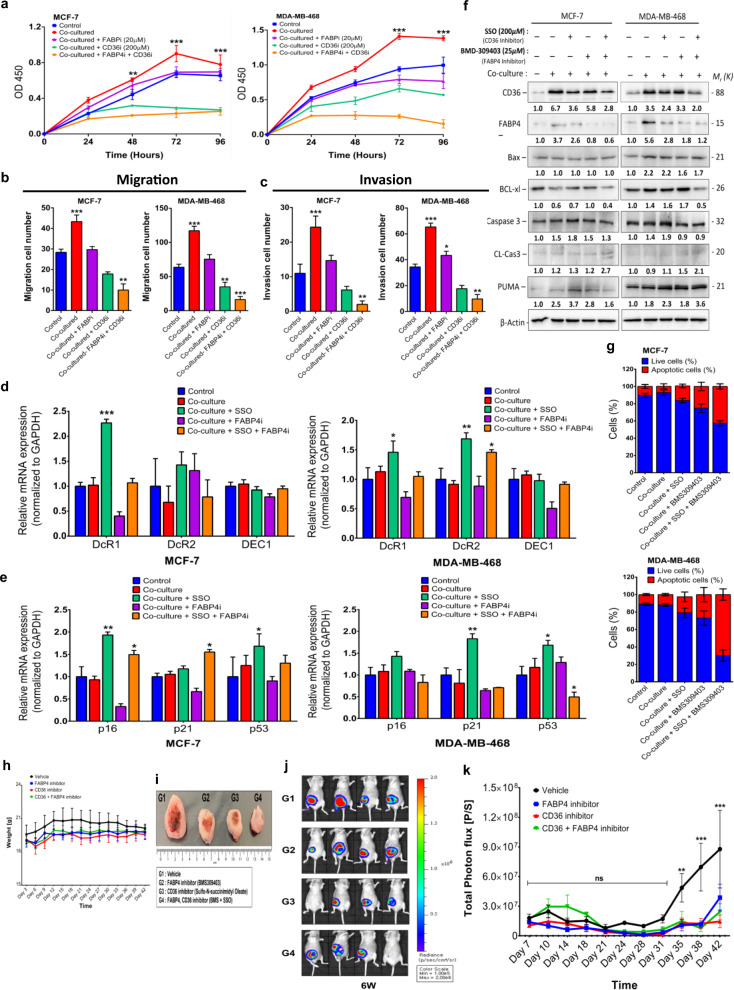
Combined inhibition of CD36 and FABP4 induces apoptosis in breast cancer cells. **A** Proliferation capabilities of breast cancer cells assessed after treatment with SSO and BMS309403 inhibitors at specific time points (0, 24, 48, 72- and 96-h). Quantitative analysis of migration (**B**) and invasion (**C**) cell numbers of MCF-7 and MDA-MB-468 cells cultured with/without adipocyte CM and treated with/without SSO and BMS309403. All results are representative of three independent experiments. **D** qRT-PCR comparing the expression of DEC1, DcR1 and DcR2 in MCF-7 and MDA-MB-468 cells cultured with/without adipocyte CM and treated with/without SSO and BMS309403﻿t. **E** qRT-PCR comparing the expression of p16, p21 and p53 in MCF-7 and MDA-MB-468 cells cultured with/without adipocyte CM and treated with/without SSO and BMS309403﻿. **F** Expression of CD36, FABP4, Bax, Bcl-xl, caspase-3, cleaved caspase-3 and PUMA in MCF-7 and MDA-MB-468 cells treated with SSO and BMS309403 assessed by western blotting. **G** Evaluation of apoptosis in breast cancer cells treated with SSO and BMS309403 using Annexin V/PI dual-labelling technique. Quantitative analysis of percentage of MCF-7 and MDA-MB-468 cells undergoing apoptosis. **H** Comparison of weight of mice from different treatment groups (Vehicle, FABP4 inhibitor, CD36 inhibitor and CD36 + FABP4 inhibitor) over time. **I** Comparison of tumour formed in mice from different treatment groups. Tumour formed in mice receiving treatment where smaller compared to vehicle group. **J** Tumour size were determined by measuring the intensity of luciferin by IVIS Lumina XRMS in vivo imaging system. **K** Plot of tumour size measurements over time. Results indicate significant inhibition of tumour formation in treatment groups compared to vehicle group. Relative mRNA expression was normalised to GAPDH. Experimental results are representative of three independent experiments. (qRT-PCR data indicate mean ± SEM; ***p < 0.001; **p < 0.01; *p < 0.05).

Various studies have reported the potential for CD36 and FABP4 to be targeted for therapy in different cancers, indicating that targeting either CD36, FABP4 or both may provide therapeutic opportunities. The observation that these proteins directly interact to regulate lipid/FFA metabolism creates an opportunity to inhibit both proteins for an enhanced effect. Thus, CD36 was inhibited, with SSO, and FABP4, with BMS309403 (Supplementary Fig. 8A), and their effects on breast cancer cells determined (Fig. 8A–C). Inhibiting either at recommended concentrations did not significantly decrease MCF-7 and MDA-MB-468 cell viability over 72 h (Supplementary Fig. 8B). However, inhibiting both CD36 and FABP4 significantly decreased the breast cancer cell viability over 72 h (Supplementary Fig. 8B). Similarly, CD36 and FABP4 inhibition significantly decreased the proliferative, migratory, and invasive abilities of breast cancer cells (Fig. 8A–C and Supplementary Fig. 8).

To understand how treatment with either SSO or BMS-309403 decreased proliferation, migration and invasion capabilities of breast cancer cells while still maintaining cell viability we assessed the expression levels of markers associated with cell senescence (DEC1, DcR1 and DcR2) and markers associated with cell cycle arrest (p21cip1, p16 and p53). In both MCF-7 and MDA-MB-468, there were unique and significant changes in the expression of cell senescence and cell cycle arrest markers (Fig. 8D, F). These findings indicate that inhibition of either CD36 with SSO or FABP4 with BMS-309403 induced incomplete senescence in MCF-7 and MDA-MB-468 cells with changes in the expression of cell senescence markers and cell cycle arrest markers (Fig. 8D, F). Combined SSO and BMS309403 treated cells treated showed apoptotic characteristics (Supplementary Fig. 8E). Hence, we determined the protein expression levels of apoptosis-associated markers. In both cells, combining SSO and BMS309403 decreased the expression of the apoptosis markers BCL-xL, with an increase in the apoptosis markers CL-Cas3 and PUMA (Fig. 8E). Assessment of the number of apoptotic cells by annexin V/PI assay showed that SSO and BMS309403 treatment increased the proportion of apoptotic cells (Fig. 8G and Supplementary Fig. 8F–I). These findings indicate that chemical inhibition of CD36 and FABP4 functions results in significant apoptosis with distinct morphological phenotypes in the breast cancer cells (Supplementary Fig. 8E). SSO treatment resulted in cell clustering, with significant lipid accumulation. Treatment with both SSO and BMS309403 resulted in clustering of cells and increased lipid content, indicating an inability to metabolise lipids resulting potentially in lipotoxicity-induced apoptosis (Supplementary Fig. 8E–I).

We further verified the potential for CD36 and FABP4 inhibitors (SSO and BMS309403, respectively) to suppress tumour growth through in vivo xenograft nude mice models. To enable physiological visualisation of tumour growth breast cancer cells were first transfected with a luciferase reporter before engrafting. Tumours were generated in female Balb/C nude mice and mice were randomly assigned into four groups of five mice each. Group 1 (G1) were treated daily with PBS (vehicle), group 2 (G2) were treated daily with 15 mg/kg dose of the FABP4 inhibitor; BMS309403, group 3 (G3) were treated with 40 mg/kg with the CD36 inhibitor (SSO) twice a week by intraperitoneal injections and group 4 (G4) were treated with a combination of BMS309403 and SSO. Although the weight of mice in the control group were slightly higher than all other groups, mice weight were not significantly different between experimental groups over the study period (Fig. 8H). We demonstrated that treatment with either BMS309403 or SSO alone decreased the rate of tumour formation and the combination of BMS309403 and SSO significantly decreased the rate of tumour formation (Fig. 8I). Measurements of tumour volume (Fig. 8I) and luciferase activity (Fig. 8J, K) revealed an initial slow response to treatment options. However, after 31 days of treatment we observe that therapy profoundly limited tumour growth. We did not observe a significant difference in tumour growth in groups that received treatment options of either BMS309403 alone, SSO alone or combination of BMS309403 and SSO. However, compared to vehicle group we observe a significant decrease in tumour growth in mice that received treatment (Fig. 8K). Taking all together these findings indicate that inhibiting both CD36 and FABP4 induces significant accumulation of unmetabolized lipid, resulting in apoptosis in breast cancer cells and chemical inhibitors of CD36 and FABP4 effectively reduces tumour growth in vivo.

## Discussion

The oncogenic properties of CD36 have been reported in breast, ovarian and hepatocellular carcinomas^14,15,18^. Contrary to decreasing breast cancer cell proliferation and aggressiveness, CD36 expression reportedly also has a tumour suppressive role. In colorectal cancer, a progressive decrease in CD36 expression reportedly leads to tumour progression from adenomas to carcinoma, with CD36 loss enhancing prognosis^19^. These contrasting reports on CD36 function may indicate its context-specific role in cancer. Thus, we extensively explored the role of CD36 in adipocyte-breast cancer cell interaction. In this study, we found that CD36 expression increased, following co-culturing breast cancer cells with hADS, and in the clinical data of breast cancer patients. IHC staining showed that a high proportion of breast tumours, with adipose tissue infiltration, have moderate to high CD36 expression. The observation of an increased CD36 expression and tumour invasiveness is consistent with finding by Zaoui et al.^20^. This result is consistent with studies by Diret et al., Manabe et al. and Fletcher et al., where CD36 expression was increased in tumour regions closely associated with adipose tissues^1,21,22^. Consistent with the context-specific CD36 expression, co-culturing breast cancer cells of distinct molecular phenotypes, with differentiated hADS, increased CD36 expression. Thus, upregulation of CD36 in response to exogenous fatty acids may be an adaption mechanism enhanced in tumourigenic breast cells.

The adipose tissue population in close/direct contact with cancer cells are commonly called cancer-associated adipocytes (CAAs). They have distinct characteristics from adipose/adipocytes not associated with cancer cells. Breast cancer tissue-infiltrated adipose tissues with breast cancer cells infiltration have significantly decreased lipid droplet size. Notably, CAAs have significantly increased expression of IL-6 and leptin. Increased CAA-released IL-6 and leptin are essential tumour growth enhancers^20,23–25^. Wang et al. recently reported that human breast adipocyte-derived leptin activates the JAK/STAT3 pathway and influences breast cancer stem cell renewal and chemoresistance^6^. These findings imply that adipocyte-cancer cell interaction increases lipolysis and resulted in an assumption of an inflammatory state. The inflamed adipocytes secrete various adipocytokines notably IL-6 and leptin that enhance carcinoma growth.

Gain- and loss-of-function studies, with stable CD36 knockouts and CD36 expression cells, showed that co-cultured breast cancer cells have phenotypic changes characteristic of EMT, i.e., dispersed, and elongated. Increased CD36 expression in hepatocellular carcinomas reportedly induces EMT and elevates FFA levels. Our results indicate that the CD36-mediated fatty acid uptake potentiates the expression of EMT transcription factors TWIST and SNAIL. Genetic ablation of CD36 inhibits fatty acid import and EMT induction, with decreased EMT-TFs and EMT-related marker expression. The correlation between increased CD36 expression and EMT markers was also present in the breast cancer cohorts. Increased CD36 expression increased the expression of the mesenchymal markers *ZEB1, ZEB2* and FN1 and decreased expression of epithelial cell markers, *CDH1, TJP3* and *MUC1*. These findings imply that in the adipocyte-rich microenvironment, increased CD36 expression enhances FFA uptake and paracrine signalling involving adipocytokines (IL-6, leptin and TGF-β) potentiates the emergence of EMT in breast cancer cells. The emergence of EMT indicates enhanced plasticity of cancer cells, which is a key contributor to the development of therapeutic resistance^17,26^.

CD36 induces EMT via the activation of oncogenic signalling pathways. Our results show that the STAT3 and ERK1/2 signalling pathways are activated, with upregulated CD36 and co-culturing. Abrogation of CD36 downregulates these signalling pathways. Activation of the STAT3 pathway is linked to both adipocyte-induced EMT and stemness in breast cancer cells. Adipocyte-induced ERK1/2 pathways have also been reported in ERα positive breast cancer cells^18^. The STAT3 pathway regulates genes mediating tumour proliferation (cyclin D1 and cyclin B), invasion (E-cadherin and FAK) and metastasis (MMP9 and TWIST)^27^. Thus, CD36-induced activation of the ERK1/2 and STAT3 signalling pathways in the adipocyte environment may account for the enhanced proliferation, migration and invasion of breast cancer cells.

Fatty acid import drives metabolic reprogramming, which shifts towards FAO as opposed to enhanced glycolysis in cancer cells^28^. In breast cancer patients, increased CD36 expression occurs, with increased expression of lipid transport receptors (LDL) and FABP4. The increased FABP4 expression in this study corroborates several studies, which have linked increased FABP4 expression to poor prognosis in several cancers, without any specific link to CD36^29–31^. We believe the correlation between CD36 and FABP4 maintains homoeostasis in the rate of fatty acid import and metabolism, ensuring that imported fatty acids are effectively shuttled to subcellular locations (mitochondria and peroxisomes). Importantly, several enzymes involved in FAO were also upregulated, with upregulated CD36 and fatty acid import. The observed increase in the expression of drug metabolism genes (*ABCB11, ABCB4, ABCG2, ABCA6, ABCC9* and *ABCD2*), with increased CD36 expression, remain unexploited. This observation has been previously highlighted by Iwamoto et al., who reported that the lipid-dependent metabolic reprogramming resulted in angiogenic drug resistance^32^. This alteration may account for the reported chemoresistance observed in breast cancer cell and the adipocyte co-culture^33^. However, further studies are required to further elucidate this relationship.

The association between CD36 and FABP4 expression has been reported by Zaoui et al. in breast cancer cells^20^. Interestingly, they observe that the ability of adipocyte to induce metabolic reprogramming occurred independent of BMI, menopausal status and mammary density. Our observation of a correlation between CD36 and FABP4 in a variety of cancers, and the direct interaction between CD36 and FABP4 offer a key insight to how adipocyte induce metabolic reprogramming. The direct interaction between CD36 and FABP4 reveals that FABP4 may mediate binding and intracellular shuttling of imported fatty acids for metabolism. We speculate that in the plasma membrane, FABP4 binds directly to CD36 and mediates fatty acid import, and in the cytosol, it shuttles fatty acid to the mitochondria and peroxisomes. This observation is particularly striking since several studies have investigate CD36 and FABP4 functions, independently. The direct interaction and interplay between these in regulating fatty acid import and metabolism requires studies that focus on how inhibiting either CD36 or FABP4 influences the other and fatty acid metabolism. This study is presently limited by the absence of an experimental in vivo model for validation; however, using human-differentiated adipocytes, compared to murine adipocytes, offers greater value and validation of the effect of SSO and BMS309403 in xenograft model offers valuable insight.

In conclusion, CD36 is a key player in adipocyte-breast cancer cell interaction. The function of CD36 is context-specific, with increased expression in adipocyte co-cultures. Increased CD36 expression activates the STAT3 signalling pathway required for adipocyte-induced EMT and stemness. STAT3 activation creates a feedforward loop for the transcriptional regulation of CD36 expression. FABP4 is a key partner for CD36. Their direct interaction regulates fatty acid import. Combined inhibition of CD36 and FABP4 may be an efficient approach, compared to independent inhibition of each. Our use of human derived adipocytes compared to the widely use murine adipocyte is an advantage. Validation of our findings in patient tissues by IHC and in patients’ data in the TCGA provides valuable insights. This study is presently limited by the absence of a suitable in vivo model to study how adipocyte influence breast cancer progression.

## Methods

### Study design

Collection of human tissues for primary adipocyte isolation and human breast cancer samples were obtained from consenting patients in accordance with guidelines and regulations approved by the Institutional Review Board of Severance Hospital, Yonsei University Health System (4-2014-0054).

### Cell culture and treatments

Breast cancer cells line MCF10A, MCF-7, MDA-MB-468, BT-483 and HCC2218 were obtained from American Type Culture Collection (ATCC, Manassas, VA, USA). Upon thawing and passaging, cells were used until passage number 20. Mycoplasma testing was done every other month, using the MycoAlert following manufacturers protocol (Lonza, Cat# LT07-318). Non-tumorigenic breast epithelial cell MCF10A cultured in DMEM supplemented with 5% horse serum, 1% penicillin-streptomycin. MDA-MB-468 and MCF-7 were cultured in DMEM mixed with F12 (DMEM/F12; Welgene) supplemented with 10% FBS and 1% penicillin-streptomycin. BT-483 and HCC2218 cells were cultured in RPMI-1640 supplemented with 20% FBS, 1% penicillin-streptomycin. Where required cells were treated with 200 μM of Sulfo-N-succinimidyl oleate (SSO), 100 μM STAT3 inhibitor (S3I-201) (Santa Cruz), 60 nM ERK1/2 inhibitor (U0126) (Santa Cruz) and 20 μM BMS-309402 (Millipore).

### Differentiation of adipocytes and collection of adipocyte-conditioned media

Primary adipocytes were isolated from subcutaneous adipose tissues as by-product of human patients with colon cancer as described by Lee et al.^34,35^ Isolated primary preadipocytes was maintained and expanded in DMEM, supplemented with 10% fatal bovine serum (FBS; Gibco^TM^, Life Technologies, Carlsbad, CA, USA) and 1% penicillin-streptomycin (Gibco^TM^). Differentiation into matured adipocytes was done using adipogenic differentiation media [DMEM with 5% FBS and 1% Penicillin-streptomycin supplemented with 0.5 mM isobutylmethylxanthine (Sigma-Aldrich, St Louis, MO, USA), 1 μM dexamethasone (Sigma-Aldrich), 1 μg/ml insulin (Sigma-Aldrich), 2 nM T3, 10 μg/ml transferrin, 1 μM rosiglitazone, 33 uM biotin and 17 uM pantothenic acid] for 7 days. Adipogenic differentiation media was replaced with adipogenic maintenance media [DMEM with 5% FBS and 1% Penicillin-streptomycin supplemented with 1 μg/ml insulin (Sigma-Aldrich)] and 10 nmol/l dexamethasone (Sigma-Aldrich) for an additional 7 days. Adipocytes are rinsed with PBS and replaced with complete media without differentiation factors and used in direct co-culture. Collection of human tissues for primary adipocyte isolation was carried out in accordance with guidelines and regulations approved by the Institutional Review Board of Severance Hospital, Yonsei University Health System (4-2014-0054), after informed consent was obtained from patients. Following full differentiation of adipocytes, maintenance media is replaced with fresh complete media after 48 h, adipocyte-conditioned media is collected, centrifuged at −4 °C. The supernatant is collected and stored at −20 °C until used.

### Co-culture

Co-culture of adipocytes and breast cancer was done by two methods. The direct co-culture procedure, adipocytes are differentiated in the bottom well of the 6-well transwell co-culture system. Breast cancer cells are subsequently seeded into the transwell inserts and cultured with adipocytes for the required period. Experiments are conducted by direct co-culture unless otherwise indicated. In the indirect-co-culture system, cell culture media of breast cancer cells is replaced with 75% adipocyte-conditioned media. All experiments requiring chemical treatment are conducted by the indirect co-culture method.

### Oil red O staining/intracellular lipid quantification

Differentiated hADS and co-cultured breast cancer cells was evaluated for their intracellular lipid content by Oil Red O staining. Cells are fixed with 4% paraformaldehyde for 20 min at room temperature (RT), rinsed with PBS and distilled water and stained for 30 min with Oil Red O. Cells are five times with water and images acquired using the Olympus BX53 microscope (Olympus Optical Co., Tokyo, Japan). Quantification of intracellular lipid content was performed after stained cells are rinsed with water and with 60% isopropanol. In total, 100% isopropanol was used to extract Oil Red O and 200 µl of extracted Oil Red O is transferred into a 96-well plate (in triplicated) and measured spectrophotometrically at 492 nm (Tecan Group limited, Männedorf, Switzerland).

### Triglyceride and glycerol content analysis

Intracellular triglyceride in adipocytes and breast cancer cells were quantified using the Cayman triglyceride colorimetric assay kit (Cayman chemical, Ann Arbor, USA) following manufacturers instruction. Results were read spectrophotometrically at 550 nm (Tecan Group limited, Männedorf, Switzerland). Intracellular glycerol of breast cancer cells and extracellular glycerol levels from adipocytes was assessed using the glycerol-free reagent kit (Sigma-Aldrich) following manufacturer’s instruction.

### Proliferation and viability assay

For breast cancer cell proliferation rate, 2500 cells were seeded into a 96-well plate for overnight. Complete media is replaced with serum free media and cultured for 12 h. After 12 h, media is replaced with adipocyte CM or complete media and cell proliferation assessed at 6, 12, 24, 36, 48, 60, 72 and 96 h, all reactions are performed in triplicate. After each time, 10 μl of CCK-8 solution (Dojindo, Kumamoto, Japan) was added to each well and incubated for 2 h and optical density was measured spectrophotometrically at 450 nm (Tecan Group limited, Männedorf, Switzerland). Cell viability after CD36 knockout or treatment with SSO, S31-201, U0126 and BMS-30940 was determined by seeding 2500 cells in 96-well plate, and cultured for 24 h, after which treatment agent is added at appropriate concentration and maintained for 72 h. Cell viabilities was evaluated at 6, 12, 24, 36, 48, 60 and 72 h using the EZ-CYTOX cell viability kit (Douzen, Seoul, Korea). Briefly, 10 μl of EZ-cytox reagent (DoGEN, Kumamoto, Korea) was added to each well and incubated for 2 h at 37 °C and absorbance measured at 450 nm spectrophotometrically (Tecan Group limited, Männedorf, Switzerland).

### Colony and spheroid formation assay

For colony formation assay co-cultured and control breast cancer cells were seeded at 1000 cells per ml in 6-well plates and incubated at 37 °C. Cells are maintained in complete or adipocyte-conditioned media for 14 days. Cell culture media was changed every 3 days. After day 14, cells are rinsed with PBS, fixed with acetic acid and methanol (1:3), and stained with 0.05% crystal violet. Images are captured and number of colonies formed counted using ImageJ software. For spheroid formation, 2500 co-cultured and control cells were mixed with DMEM containing 0.35% soft agar (Difco, Mubai, India) and plated in a six-well plate coated with 0.6% soft agar. Adipocyte CM or complete media is added to the plate after every 3 days. After 14 days, the number of spheroids formed in three random microscopic fields were counted, and the images were captured using the lionheart FX microscope (BioTek Instruments Inc. Winooski, VT, USA).

### Wound healing, migration and invasion assay

Wound healing assay for co-cultured and control MDA-MB-468 and MCF-7 cells are seeded at 5 × 10^5^ into six-well plates and cultured to 90% confluence, complete media is replaced with serum free media and cells are cultured overnight until a monolayer is formed. A sterile 100 μl pipette tip us used to make a linear scratch and dissociated cells washed with cell culture media and cultured with complete media or adipocyte-conditioned media. Images of was acquired at 0 and 48 h at ×100 magnification. The captured images were analysed using ImageJ software. Breast cell migration and invasion was assessed using corning transwell insert (8-μm pore size; Corning Inc.) without matrigel (Migration assay) and with 1.0 mg/ml matrigel (Invasion assay) (BD Biosciences, Bedford, MA, USA). Co-cultured and control cells were seeded at 5 × 10^4^ cells in 300 μl of serum free media into the upper chamber of the transwell plate with 10% FBS media in the bottom chamber. Cells were cultured for 24 h for migration and 48 h for invasion. After removal of non-invading cells, invading cells were fixed and stained by the Diff-Quik kit (Sysmex, Seoul, Korea) and the number of migrating and invasion cells was counted under a microscope in five random fields of each membrane at ×100 magnification. All experiments were repeated at least three times.

### CD36/CD44 flow cytometry and annexin V/PI assay

To determine the proportion of CD36/CD44 expressing cells, cells co-cultured and control cells were harvested and resuspended to ~1–5 × 10^6^ cells/ml in ice cold PBS. Cells are fixed with 4% formaldehyde for 15 min at RT. Cells are resuspended in 100 μl BSA/PBS and incubated with primary antibodies against CD36 and CD44 and for 1 h at RT. Cells are rinsed 3X and resuspended in ice cold PBS. Cells are incubated with Alexa Fluor 488 and Alexa Fluor 647-conjugated secondary antibody for another 30 min, rinsed and analysed. Cells are initially selected for FSC-A/SSC-A gating followed by SSC-H/CD44-FITC gating, CD36-APC/FSC-H gating and CD36-APC/CD44-FITC positive cell gating. To determine the proportion of apoptotic cells, cells treated with specific inhibitors were seeded at 4 × 10^5^ cells and stained using Alexa Fluor 488 annexin V/Dead cell apoptotic kit (Invitrogen, Paisley, UK) following manufacturers instruction. Apoptotic cells were detected by flow cytometry using BD FACSAria™ III (BD Biosciences, San Jose, USA).

### Protein extraction and western blot

Protein were extracted from cells using RIPA buffer (GenDEPOT, TX, USA) containing 1% protease inhibitor and 1% phosphatase inhibitor cocktail (GenDEPOT) as lysis buffer. In total, 20 μg of extracted proteins is separated by SDS-PAGE and transferred to a nitrocellulose membrane (GE Healthcare, Chalfont St Giles, UK). Immunoblotting was performed as previously described by Gyamfi et al.^4^ List of all primary antibodies and dilutions used are indicated in Supplementary Table 6. All gels and blots were derived from the same experiment and were processed in parallel.

### RNA extraction and quantitative real-time PCR

Total RNA from co-cultured and control cells were extracted was isolated using the RNeasy Kit (Qiagen, Valencia, CA, USA) following manufacturer’s instruction. A one-step real-time PCR was performed with 50 ng of RNA using the TOPreal One-step RT-qPCR kit (SYBR Green with high ROX) (enzynomics, KR) according to the manufacturer’s instruction and analysed with the StepOne Plus Real-time PCR system (Applied Biosystems, Foster City, CA, USA). All reactions were performed in triplicate; with the housekeeping gene glyceraldehyde 3-phosphate dehydrogenase as an internal control mRNA. All primers were initially evaluated for efficiency using the Relative standard curve and the relative gene expression evaluated by comparative CT method (2^−∆∆CT^). Amplification primers are listed in Supplementary Table 2.

### Immunofluorescence

Immunofluorescence was performed as previous described by Gyamfi et al. Briefly, cells are seeded on cover slide placed in co-culture insert and co-cultured with adipocytes for 48 h. Cells was rinsed in PBS and fixed with 4% paraformaldehyde, permeabilized with 0.2% Triton X-100 and stained with appropriate primary antibodies. For double staining experiments, antibodies were diluted together and incubated with cells overnight at 4 °C. Goat Anti-Rabbit IgG (Alexa Fluor 647) and Goat Anti-mouse IgG (Alexa Fluor 488) antibodies (Abcam) were used as secondary antibodies. Counter staining of cell nuclei was performed using DAPI (Invitrogen, Carlsbad, CA, USA). Stained cells were visualised using the ZEISS LSM 710 microscope (ZEISS, Germany). Antibodies used included mouse anti-E-cadherin 1:500 (Cell Signalling, Danvers, MA, USA), rabbit anti-Vimentin 1:500 (Proteintech).

### Luciferase reporter assay

For reporter assay, mock-CD36, CD36-expressing and CD36-knockdown cells are transiently co-transfected with cignal-STAT3 reporter plasmid or cignal-PPAR reporter plasmid (Cignal Lenti-PPAR Reporter, QIAGEN, Hilden, Germany). Promoter activity is measured at specific time points (0, 1, 6, 12, 24 and 48 h). Promoter activity was determined using the Promega Dual-Luciferase reporter assay system (Promega corporation, Madison, USA) following manufacturers instruction and luciferase activity measured in the Tecan™ microplate-Luminometer (Tecan Group Limited, Männedorf, Switzerland). The constitutively expressed non-inducible Renilla luciferase activity served as internal control for normalising transfection efficiencies.

### Immunohistochemistry

Formalin fixed tissues sections are deparaffinized by passing sections through xylene, followed by a series of decreasing concentration of alcohol. Antigen retrieval was performed by immersing tissue sections in 10 mM pre-heated citrate buffer (pH 6.0) and microwaved for 5 min and cooled at RT for 20 min. Endogenous peroxidase activity was quenched by immersing samples in 3% H_2_O_2_ for 15 min and incubated with anti-CD36 primary antibody diluted at 1:1000 and incubated at 4 °C overnight. The slides are developed using the EnVision Dual Link System-HRP kit (DAKO) according to the manufacturer’s instruction. All IHC results were examined and scored from 1 to 4 based on their expression intensity and percentage of stained cell population by two independent scientists.

### Co-immunoprecipitation assay

Co-IP assay was performed using the Thermo Scientific co-IP kit following the manufacturer’s protocol. Primary antibodies (2 μg of CD36) and (2 μg of FABP4) were immobilised for 2 h using AminoLink Plus coupling resin at 4 °C. Cell lysate (500 μg of total protein) was pre-cleared by incubating with control agarose resin for 1 h at 4 °C. Antibody coupled resin was then incubated with pre-cleared protein lysate overnight at 4 °C. Resins were washed and proteins eluted. Eluted protein was separated on SDS-PAGE and analysed by western blot.

### Identification of STAT3 binding sites and chromatin immunoprecipitation (ChIP) assay

Two transcription factor binding site prediction tools, rVista (http://rvista.dcode.org) and CONTRA v2 (http://www.dmbr.ugent.be/prx/bioit2-public/contrav2/index.php), and NCBI sequence finder tool was used to identify putative STAT3 transcription factor binding sites present in the CD36 promoter. Chromatin was isolated from cells after cross-linked by 1% formaldehyde, using the Chromatin Extraction Kit (ab117152, Abcam-USA), following manufacturers instruction. Isolated chromatin was sonicated for 10 min and chromatin immunoprecipitation was done with the One-Step Chip kit (Abcam, ab117138). Antibody recognising pSTAT3 (abcam, dilution 1:50) was used as the pull-down was used for the ChIP assay. The purified DNA was analysed by real-time PCR with a PCR kit (Takara Bio Inc., Japan) according to the manufacturer’s protocols. The primers for ChIP-qPCR are provided in Supplementary Table 3.

### Seahorse metabolic assay

Cells are cultured with/without adipocytes and seeded evenly at 40,000 cells/well in the XF24 cell culture plate and allowed to attach for 24 h. Cells were assessed with the XF cell energy phenotype test, XF cell mito-stress test, XF mito-fuel flex test and XF palmate-BSA FAO test (Agilent Technologies, UK) following the manufacturers protocol. After each assay, floating cells are recovered, centrifuged and attached cells lysed with RIPA buffer. Protein concentration was determined using Pierce BCA protein assay kit (Thermo Fisher Scientific) for normalisation.

### Transfection and generation of stable cell lines

The full-length human CD36 (hCD36) cDNA was amplified with PCR using specific primers and subcloned into the EcoR1 and XbaI sites of the pLVX-EF1a-IRES-Puro (clontech) to generate the pLVX-EIP-CD36 plasmid. Lentivirus expressing hCD36 was produced by transfecting HEK293T cells (ATCC) with plasmids; pLVX-EIP-CD36, psPAX2 (Addgene, plasmid #1260) and pMD2.G (Addgene, plasmid #12259) using DharmaFECT-Kb transfection reagent (GE Healthcare). Virus conditioned media was harvested after 48 h and cleared with filtration through 0.44 nm pore before use. To generate stable CD36-expressing cells, cells were cultured with viral conditioned media in 1:1 ration containing polybrene for 48 h. Monoclonal CD36 expression cells were selected with 10 µg/ml puromycin over 14 days. To generate stable CD36-knockout MCF-7 and MDA-MB-468 cells, we selected three CRISPR guide RNA targeting CD36 cloned into the pLentiCRISPRv2 plasmid. Lentivirus expressing CD36 guide-RNAs was produced by transfecting HEK293T cells with pLentiCRISPRv2 plasmid, psPAX2 (Addgene, plasmid #1260) and pMD2.G (Addgene, plasmid #12259) using DharmaFECT-Kb transfection reagent (GE Healthcare). Virus conditioned media was harvested after 48 h and cleared with filtration through 0.44 nm pore before use. To generate stable CD36-knockout cells, MCF-7 and MDA-MB-468 cells were cultured with viral conditioned media in 1:1 ratio containing polybrene for 48 h. Stable monoclonal CD36-knockout cells were selected using 10 µg/ml puromycin over 14 days. CRISPR guide RNA sequences are provided in Supplementary Table 2, CD36 plasmid DNA amplification primers are provided in Supplementary Table 4 and CD36 CDS amplification primers are provided in Supplementary Table 5.

### GST pull-down assay

For bacteria GST pull-down assay, full human CD36 cDNA was cloned into the pGEX-4T-3 plasmid and used to transform a BL21 bacteria. CD36-GST-tagged protein expression was induced with 1 mM IPTG and GST-tagged CD36 purified by the GST SpinTrap colum (GE Healthcare, UK). For GST-CD36 pull-down assay, proteins from control CD36-expressing and CD36-knockout MCF-7 and MDA-468 breast cancer cells were mixed with GST-tagged CD36 proteins and incubated at 4 °C for 4 h with rotating. Pull-down proteins eluted with elution buffer and 1/20 of the extract was subjected to SDS/PAGE and immunoblotting performed using anti-FABP4 antibody. For mammalian GST assay, CD36 cDNA was cloned into the pcDNA3.1 + N-GST(Thrombin) plasmid and transfected into breast cancer cells.

### In vivo xenograft mouse model

All mouse studies were approved by the Institutional Animal Care and Use Committee of Yonsei University, South Korea. Seven-week-old female Balb/C Nude mice were purchased from Orient bio, South Korea. Mice were housed for a week to allow for additional adjustment under semi-pathogen free facility and were fed sterilised water and food. MDA-MB-231 cells were infected by firefly luciferase lentivirus and selected by puromycin. In total, 3 × 10^6^ cells diluted in 80 μl of PBS were injected orthotopically into 8-week-old mice. Mice were randomly assigned to experimental groups of five mice each and housed under the same conditions. Group 1 (G1) were the control group and received no treatment, group 2 (G2) were treated with the FABP4 inhibitor: BMS309403, group 3 (G3) were treated with the CD36 inhibitor; SSO and group 4 (G4) were treated with a combination of BMS309403 and SSO. FABP4 inhibitor (BMS309403) were purchased from Selleckchem and CD36 inhibitor (SSO) were purchased from Cayman. Following manufacturer’s instructions, BMS309403 were dissolved in 10% 1-methyl-2-pyrrolidone, 5% cremophor, 2% ethanol and 83% PBS. SSO were dissolved in 10% DMSO, 40% PEG, 5% Tween-80 and 45% PBS. In total, 15 mg/kg dose BMS309403 were fed daily by oral gavage and 40 mg/kg dose SSO were Intraperitoneally injected into mice twice in a week. The weight and survival condition of mice were measured on every 3rd day. Tumour size estimation was done by measuring the intensity of luciferin in each mouse twice in a week by IVIS Lumina XRMS In vivo imaging system, PerkinElmer. To validate intensity of luciferin XenoLight D-luciferin—potassium salt Bioluminescent Substrate, PerkinElmer were dissolved in PBS. In total, 150 mg/kg Luciferin were Intraperitoneally injected 10 min before estimated intensity. Signals of luciferin of each mouse were validated by Living image software, PerkinElmer. After the 42 days, mice were sacrificed, and tumour nodules excised and measured.

### Data availability and EMT score calculation

The referenced TCGA data in the study are available in a public repository from the cBioPortal website (https://cbioportal.org), Morpheus online tool (https://software.broadinstitute.org/morpheus/), GEPIA website (http://gepia.cancer-pku.cn) and UALCAN website (http://ualcan.path.uab.edu/cgi-bin/ualcan-res.pl). Gene expression profile for EMT markers, fatty acid transmembrane transporters, fatty acid transporter activity were done using gene set data available at the molecular signature database (MsigDB) website (http://software.broadinstitute.org/gsea/msigdb/genesets.jsp?collection=C5)^36,37^. A list of specifically used gene set are listed in Table 3. EMT score was calculated using the formula [EMT score = Sum of mesenchymal gene expression (CDH2, FN1, SNAI1, SNAI2, TWIST1, TWIST2, VIM, ZEB1, ZEB2) − Sum of epithelial gene expression (CDH1, CLDN4, CLDN7, MUC1, TJP3)]^15^. Hierarchical clustering, heatmap visualisations and correlation matrix visualisations were generated using the Morpheus analysis platform. Clusters were obtained using the average linkage method with 1-Pearson’s correlation coefficient as the distance metric. Gene sets used in various heatmaps and correlation matrixes are presented in Table 4.

### Statistical analysis

All experiments were performed three times independently under similar conditions. Results are shown as the means and the error bars represent the standard error of the mean (SD), unless stated otherwise. Data were analysed, and graphs plotted with GraphPad Prism version 6 software (GraphPad Inc.). *p* values were calculated by Student’s *t* test was used to compare differences between two groups and multiple analysis was performed using analysis of variance (ANOVA). Multiple analysis of groups was checked for after ANOVA using Bonferroni’s multiple comparison test. *p* values of statistical significance are represented as *p ≤ 0.05, **p ≤ 0.001 and ***p < 0.0001.

### Reporting summary

Further information on research design is available in the Nature Research Reporting Summary linked to this article.

## Supplementary information

Supplementary Information

Reporting Summary

## Data Availability

All data generated or analysed during this study are included in this published article (and its Supplementary Information files). Breast cancer patient data are available on the online portal https://software.broadinstitute.org/morpheus/. Gene set data were obtained from MSigDB and gene set name is referenced in the Supplementary Data. Datasets supporting the figures and tables in this published article are all from publicly available dataset and have been referenced in the study. Patient data for IHC are not provided to protect patient privacy but can be accessed from the corresponding author on request. All the uncropped western blots generated during this study are available in Supplementary Fig. 7.

## Code availability

Bioinformatics analyses in this study were conducted with open-source software.
