# Supplementary material for: Interaction between CD36 and FABP4 modulates adipocyte-induced fatty acid import and metabolism in breast cancer
Source: NPJ Breast Cancer. 2021 Sep 24;7:129. doi: 10.1038/s41523-021-00324-7 (PMC8463699; doi:10.1038/s41523-021-00324-7)

Supplementary Data to

Interaction between CD36 and FABP4 modulates adipocyte-induced fatty acid import and metabolism in breast cancer.

Jones Gyamfi, Joo Hye Yeo , Doru Kwon, Byung Soh Min, Yoon Jin Cha, Ja Seung Koo, Joon Jeong, Jinu Lee, Junjeong Choi

Supplementary Tables:

Supplementary table 1: Primer Sequences for qRT-PCR

PRIMER	SEQUENCE	SIZE
ADIPOCYTOKINE FACTORS		
CXCL12	*F: GGTCAGACCGCGGTGACTTC*<br>*R: AGGTACCTGGGGAGGGGAGA*	144
TGF-B	*F: CTGCAATCTCCGCCTCCTGG*<br>*R: AAAAAGAGGCCAGGCGCAGT*	230
IL-6	*F: CCACTCACCTCTTCAGAACGAAT*<br>*R: TTGGAAGCATCCATCTTTTTCA*	174
LEPTIN	*F: TGGTGAGGGAGGGTGGAAGG*<br>*R: ATGGGGTGGAGCCCAGGAAT*	188
IL-1B	*F: TCATCCACCTCGGCTTCCCA*<br>*R: GGAGAGAGCGAGGGAGGGAG*	191
TNF-A	*F: CCCAGGGGACCTCTCTCTAATC*<br>*R: ATGGGCTACAGGCTTGTCACT*	272
ADIPONECTIN	*F: TGGTGAGAAGGGTGAGAA*<br>*R: AGATCTTGGTAAAGCGAATG*	221
ADIPOCYTOKINE RECEPTORS		
TGF-B-R	*F: AGGATTGCTGGAGCCTGGGA*<br>*R: TGCCAGTGCTGGAAAGCAGG*	263
IL-6R	*F: GAGGGCTTCTGCCATTTCTGAG*<br>*R: CCAGGTTCAGCTGACAACAAACA*	69
LEPTIN-R	*F: AGGAAGCCCGAAGTTGTGTT*<br>*R: TCTGGTCCCGTCAATCTGA*	100
ADIPO-R1	*F: CTTCTACTGCTCCCCACAGC*<br>*R: GACAAAGCCCTCAGCGATAG*	174
ADIPO-R2	*F: ATAGGGCAGATAGGCTGGTTGA*<br>*R: GGATCCGGGCAGCATACA*	79
FATTY ACID TRANSPORTER		
CD36	*F: TCAAGTCCAGAAGGGCGTGC*<br>*R: GCTTGGGCTCAAGGGTAGTGG*	156
FABP4	*F: TGCCACCAGGAAAGTGGCTG*<br>*R: ACTCTCGTGGAAGTGACGCC*	300
FATP4	*F: CGGTTCTGGGACGATTGTAT*<br>*R: AACCTGGTGCTGGTTTTCTG*	391
EMT MARKERS		
SNAIL	*F: CACCTCCAGACCCACTCAGAT*<br>*R: CCTGAGTGGGGTGGGAGCTTCC*	489
MMP9	*F: AGGTTCGGCCTTTTCTGCCC*<br>*R: CCCATCACCGTCGAGTCAGC*	219
TWIST	*F: CCACGCTGCCCTCGGACAAG*<br>*R: CCAGGCCCCCTCCATCCTCC*	189
N-CADHERIN	*F: AGCCAAGGGAATTCAGCACCC*<br>*R: ATGGTACCGGCATGAAGCCC*	135
E-CADHERIN	*F: CACTTGAGCCCAGGGGGTTG*<br>*R: GGATTACAGGAGCCCGCCAC*	277
STEM CELL MARKER		
CD44	*F: TCCTTTCTGCACTGCGGGAG*<br>*R: TGAGGCTGCTGTGACCATGC*	266

CD133	*F: CTCCCCAGAATGGCTGCCTG*<br>*R: TGGCATTCCCGGAAGGGAGA*	206
OCT4	*F: AAGCTTGCCCTTGTCACCCC*<br>*R: AGTGTGGGTTTCGGGCACTG*	149
SOX2	*F: GGGAAATGGGAGGGGTGCAAAAGAGG*<br>*R: TTGCGTGAGTGTGGATGGGATTGGTG*	151
SIGNALING TARGETS		
STAT3	*F: TGAGACTTGGGCTTACCATTGGGT*<br>*R: TCTTTAATGGGCCACAACAGGGCT*	174
ERK1	*F: CAAGACCTGCCTGGGCAACA*<br>*R: CTGCAGCCCGGATGACAGAG*	265
ERK2	*F: GCACCAGACCTACTGCCAGAGA*<br>*R: TGCTCGATGGTTGGTGCTCG*	111
METABOLIC MARKERS		
CPT1A	*F: CAGGAAGTTGCACCCTGGCA*<br>*R: ACTACACTCCAGCCTCGGCA*	215
ATGL	*F: ACCAGCATCCAGTTCAACCT*<br>*R: ATCCCTGCTTGCACATCTCT*	1017
AMPK	*F: CCACCATCATGCCTGGCTGT*<br>*R: CACTGGGAGGGAAAGGCACA*	139
FASN	*F: GCCATTCGGCCTGAAGGTGT*<br>*R: CCTCCAGTAGGCAGCGAGGA*	235
ACACA	*F: CGGAAGGGACAGTAGAAATCA*<br>*R: AGTCGCTCAGCCAAGTGGA*	94
ACLY	*F: CTACCACCCAGAGCACCCCT*<br>*R: GCTTCCTCCCTGCAACACACA*	122
PPARA	*F: AAGAGGTCGGACATGGGCCT*<br>*R: AGTGTGGTGGCGTGACCTTG*	252
PPARB	*F: TGGGGTGGAAGTAGGGGAGC*<br>*R: ATCCGCTGCATCATCTGGGC*	246
PPARG	*F: CGAGAAGGAGAAGCTGTTGG*<br>*R: TCAGCGGGAAGGACTTTATG*	122
SENESCENCE MARKERS		
DEC1	*F: AGCACGGAGACCTACCAGGG*<br>*R: GCCGGTGCGGCAATTTGTAG*	546
DCR1	*F: GCTTACTCTGCCACCACTGCC*<br>*R: CTGCTGGACACTCCTCCCCC*	100
DCR2	*F: TCCTGGGGATGCTTGCCTCT*<br>*R: CATGAACGCCGCCGGAAAAG*	187
P16	*F: CGGTGCCTCACGCCTTGTAA*<br>*R: CCAGGCTGGAGTGAAGTGGC*	253
P21	*F: AGGTGGACCTGGAGACTCTCA*<br>*R: TCCTCTTGGAGAAGATCAGCCG*	299
P53	*F: GAGCTGAATGAGGCCTTGGA*<br>*R: CTGAGTCAGGCCCTTCTGTCTT*	1069
GAPDH	*F: ACCCACTCCTCCACCTTTGA*<br>*R: CTGTTGCTGTAGCCAAATTCGT*	205

Supplementary table 2: CD36 CRISPR guide RNA sequences.

gRNA	gRNA target sequence	Vector
CD36 CRISPR Guide RNA or crRNA 1	*CTCACTCACCTGTACGTATA*	pLentiCRISPR v2
CD36 CRISPR Guide RNA or crRNA 2	*ACCTTTATATGTGTCGATTA*	pLentiCRISPR v2
CD36 CRISPR Guide RNA or crRNA 3	*TAGCAAGTTGTCCTCGAAGA*	pLentiCRISPR v2
Negative control (Scambled)	*AGTCTATCGATATTATTCGT*	pLentiCRISPR v2

Supplementary table 3: ChiP primer sequence and targeted GAS-sequences.

Primer No:	GAS-sequence	Promoter Location	Primer sequence
Primer 1	TTCTAGGAA	1. 162 – 170	*F: GTGTGTATTTCCTGTGTGTTTCCTGA*<br>*R: TCAGACACATCTTGGGCCAGTG*
Primer 2	TTCTAGGAA	1. 8122 – 8130	*F: GTGTGTATTTCCTGTGTGTTTCCTGA*<br>*R: TCAGACACATCTTGGGCCAGTG*
Primer 3	TTCCTGTAA	1. 16460 – 16468	*F: CCTGTGTGTTTCCTGAAAAGGAAAGTT*<br>*R: TCAGACACATCTTGGGCCAGTG*
Primer 4	TTACTTGAA	1. 24517 – 24525	*F: TGTGCTCTGTATGTCTCACCTCA*<br>*R: ACCCTCTCAGTAAATGGCTACCAA*
Primer 5	TTCCTGAAA	1. 37860 – 37868	*F: GGGACTTGTTTCTAGAAGGATCCCAA*<br>*R: AACCCATGGGCTCCACAAGT*
Primer 6	TTCCTGTAA	1. 43935 – 43943	*F: CCTGTGTGTTTCCTGAAAAGGAAAGTT*<br>*R: TCAGACACATCTTGGGCCAGTG*

Supplementary table 4: CD36 plasmid cDNA amplification primers.

Primer	Amplification sequence
EcoR1 CD36 U1	*TTTTTGAATTCCACCATGGGCTGTGACCGGA*
Xba1 CD36 L1	*TTTTTTCTAGATTATTTTATTGTTTTCGATCTGCATG*

Supplementary table 5: CD36 CDS amplification primers.

Primer	Amplification sequence
Primer -F	AGGTCGACTCTAGAGGATCCCGCCACCATGGGCTGTGACCGGAACTGTGG
Primer - R	TCCTTGTAGTCCATACCGGTTTTTATTGTTTTCGATCTGCATGC

Supplementary table 6: Antibodies used in the study

Antibody	Company	Catalog No.	Dilutions
**CD36**	Proteintech	18836-1-AP	WB: 1: 1000<br>FC: 1: 100<br>IP: 1:200<br>IHC: 1: 1000
**Vimentin**	Proteintech	60330-1-Ig	WB: 1:1000<br>IF: 1: 200
**ZEB1**	Sigma-Aldrich	HPA027524	WB: 1:500
**E-cadherin**	Cell Signalling	3195	WB: 1:1000<br>IF: 1:200
**CD44**	Cell Signalling	3570	WB: 1:1000<br>FC: 1:100
**CD133**	Abcam	Ab19898	WB: 1:1000
**ALDH**	BD	611195	WB: 1:500
**AKT**	Cell Signalling	9272	WB: 1:1000
**pAKT (ser473)**	Cell Signalling	9271	WB: 1:1000
**pAKT (Thr308)**	Cell Signalling	5106	WB: 1:1000
**ERK1/2**	Cell Signalling	9102	WB: 1:1000
**pERK1/2 (Thr202/Tyr204)**	Cell Signalling	9191	WB: 1:1000
**P38-MAPK**	Santa cruz	Sc-728	WB: 1: 1000
**pP38-MAPK**	Cell Signalling	9211	WB: 1: 1000
**SMAD 2/3**	Cell Signalling	3102	WB: 1: 1000
**pSMAD2/3**	Cell Signalling	8828	WB: 1: 1000
**STAT3**	Cell Signalling	9139	WB: 1: 1000
**pSTAT3 (Y705)**	Abcam	Ab76315	WB: 1: 1000<br>CHIP: 1: 20
**AMPK**	ThermoFisher	MA5-15815	WB: 1: 1000
**pAMPK (Thr172)**	ThermoFisher	44-1150G	WB: 1: 1000
**ACC**	Abcam	Ab70246	WB: 1: 1000
**pACC (Ser79)**	Cell Signalling	11818	WB: 1: 1000
**ATGL**	Abcam	Ab207799	WB: 1: 1000
**FASN**	Abcam	Ab128856	WB: 1: 1000
**PPAR-a**	Santa Cruz	Sc-398394	WB: 1: 500
**PPAR-b**	Santa Cruz	Sc-74440	WB: 1: 500
**PPAR-g**	Cell Signalling	2435	WB: 1: 1000
**FABP4**	Santa Cruz	Sc-271529	WB: 1: 1000
**BAX**	Cell Signalling	2772	WB: 1: 1000

**BCL-xL**	Cell Signalling	2762	WB: 1: 1000
**Caspase 3**	Cell Signalling	9662	WB: 1: 1000
**CL-Cas3**	Cell Signalling	9661	WB: 1: 1000
**PUMA**	Santa Cruz	sc-374223	WB: 1: 500
**GST**	Abcam	Ab19256	WB: 1: 5000
**AlexaFluor 647**	abcam	Ab150083	FC: 1:2000<br>IF: 1: 1000
**Alexafluor 488**	abcam	Ab150077	FC: 1: 2000<br>IF: 1: 2000
**IgG (Mouse)**	Santa cruz	Sc-2005	WB: 1: 2000
**IgG (Rabbit)**	Santa cruz	Sc-2004	WB: 1: 2000
**a-lamin**	Santa Cruz	Sc-518013	WB: 1: 1000
**Actin**	Santa Cruz	Sc-47778	WB: 1: 1000

Supplementary table 7: MSigDB gene set list used in the study

**MSigDB gene set**	**Reference figure**
GO_LIPID_METABOLIC_PROCESS	Figure 6a
GO_FATTY_ACID_TRANSMEMBRANE_TRANSPORT	Supplementary figure 7b
GO_FATTY_ACID_TRANSMEMBRANE_TRANSPORTER_ACTIVITY	Supplementary figure 7b
GO_FATTY_ACID_BINDING	Supplementary figure 7b

Supplementary Figures:

Supplementary figure 1

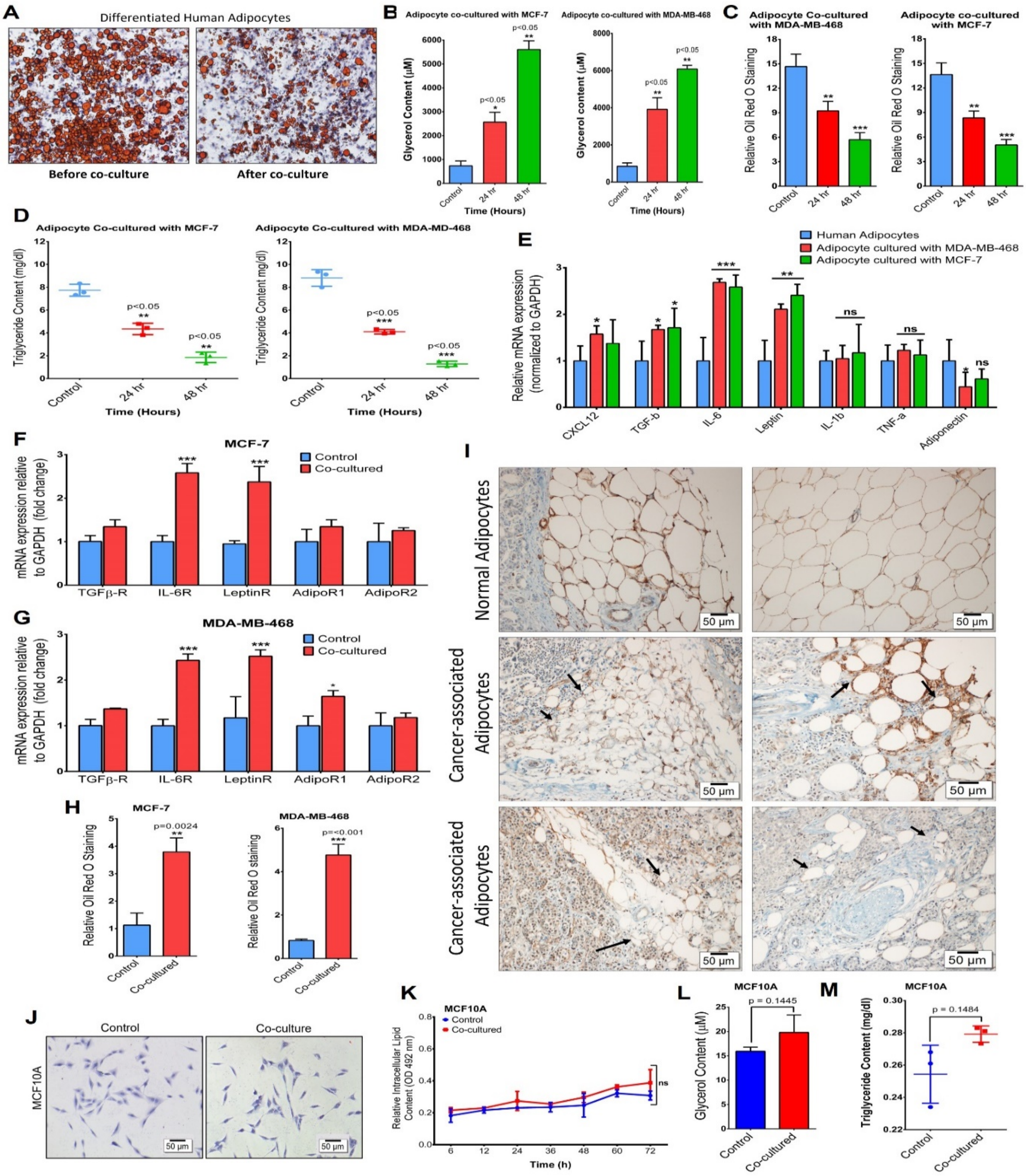

Supplementary figure 1: CD36 is key in fatty acid import in breast cancer. A) Representative image of oil red O staining in differentiated human adipocytes (hADs) before and after co-culture with breast cancer cells. B) Quantification of intracellular glycerol content in hADs after co-culture with MCF-7 and MDA-MB-468 cells. C) Quantitative estimation of accumulated fatty acid content in hADs after co-culture with MCF-7 and MDA-MB-468 cells at specific time points (24 and 48 hours). D) Quantification of intracellular triglyceride content in hADs after co-culture with MCF-7 and MDA-MB-468 cells. (Data indicate mean ± SD; ***$p < 0,001$; **$p < 0.01$; *$p < 0.05$). E) Comparison for the

expression of adipocytokine genes (CXCL12, TGF-β, IL-6, Leptin, IL-1B, TNF-a and Adiponectin) from adipocytes co-cultured with MCF-7 and MDA-MB-468 breast cancer cell. F and G) Comparison for the expression of cognate receptors for adipocytokines (TGF-βR, IL-6R, Leptin-R, AdipoR1 and AdipoR2) in MCF-7 (F) and MDA-MB-468 (G) cells cultured with/without adipocytes. Relative mRNA expressions were normalized to GAPDH, (Data are mean ± SEM., ***p < 0,001; **p < 0.01; *p < 0.05, n=3). H) Quantification for the rate of fatty acid accumulation after oil red O staining in MCF-7 and MDA-MB-468 cells after co-culture with hADs. I) Representative IHC images of CD36 expression in cancer-associated adipocytes (CCA), lipid droplet sizes in adipocytes shrunk after co-culture with breast cancer cells. J) Representative image of oil red O staining in non-tumorigenic breast epithelial cells (MCF10A) co-cultured with adipocytes for 48-hours. K) Quantification for the rate of fatty acid accumulation in MCF10A cells cultured with/without human adipocytes at specific time points (6, 12, 24, 36, 46, 60 and 72 hours). L) Quantification of intracellular glycerol content in co-cultured MCF10A. M) Quantification of intracellular triglyceride content in MCF10A cells cultured with/without adipocytes.

Supplementary figure 2

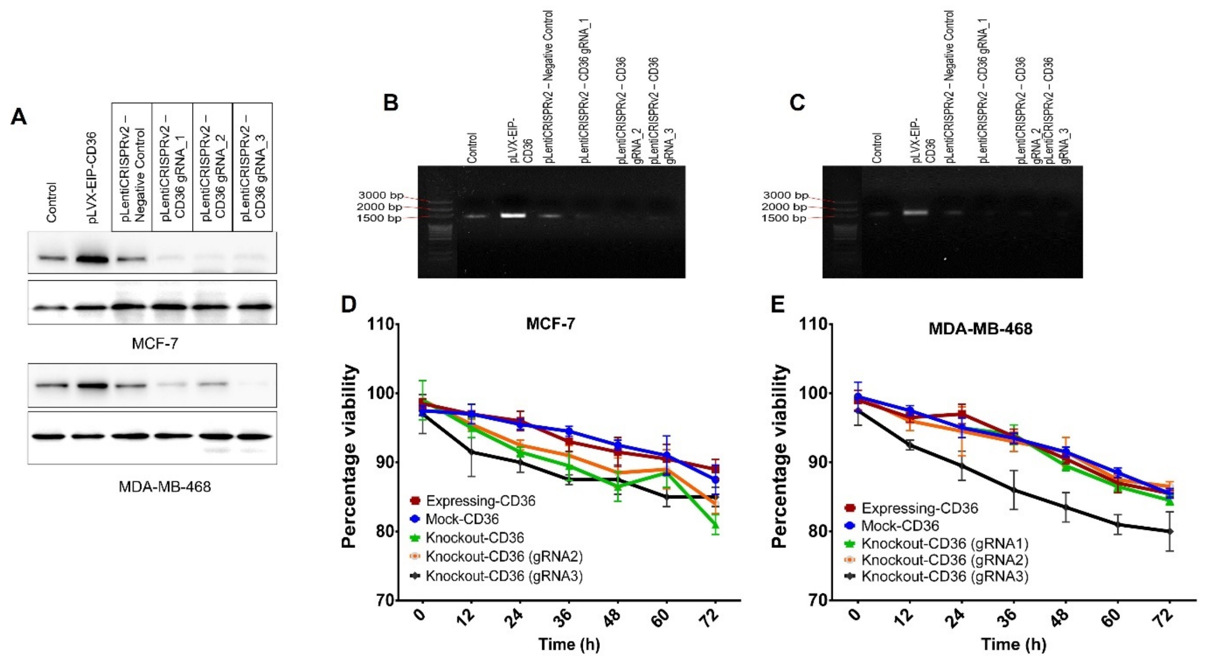

Supplementary Figure 2: Validation of CD36 ablation and upregulation. A) Western blot images for CD36 expression in breast cancer cells after genetic modification. B and C) Validation of CD36 knockout and overexpression by PCR amplification. D and E) Evaluation of CD36 ablation and CD36 upregulation on the viability of MCF-7 and MDA-MB-468 breast cancer cells co-cultured with adipocytes for 72hr. Results are representative of 3 independent experiments. (Quantification data indicate mean ± SD; ***p < 0,001; **p < 0.01; *p < 0.05).

Supplementary figure 3

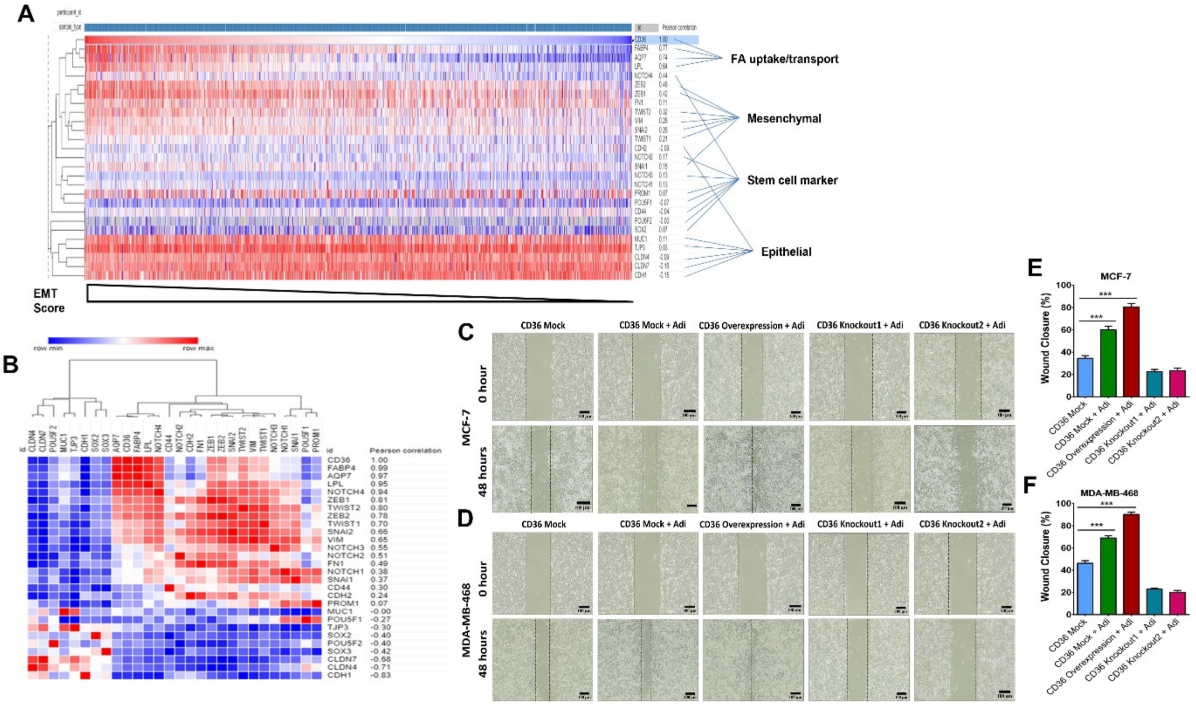

Supplementary figure 3: CD36 upregulation enhances EMT and stemness in adipocyte co-cultured breast cancer cells. A) Heatmap showing relative expression levels of fatty acid uptake genes and EMT genes in the TCGA breast cancer dataset. Gene clusters (vertical axis) were obtained by hierarchical clustering and samples (horizontal axis) were ordered according to their EMT score. B) Correlation matrix heatmaps showing the association between mRNA expression z-scores (TCGA) of fatty acid transporters and EMT markers. C and D) Evaluation of the effect of CD36 ablation on cell motility in MCF-7 and MDA-MB-468 cells co-cultured with adipocytes. Representative images of MCF-7 (C) and MDA-MB-468 (D) wound healing assay (magnification, x100). E and F) Quantitative analysis of wound closure rate (%) in MCF-7 (E) and MDA-MB-468 (F) cells. All results are representative of 3 independent experiments. (Data indicate mean ± SD; ***p< 0.001; **p<0.01; *p<0.05).

Supplementary figure 4

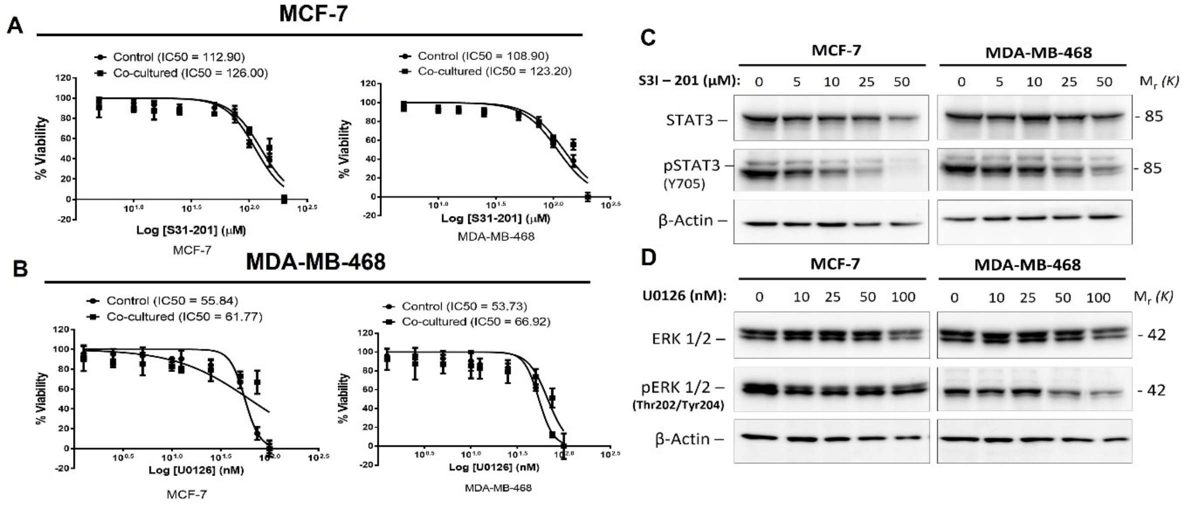

Supplementary figure 4: Validation of the effect of S3I-201 and U0126 on breast cancer cells. A) Cell viability curves for the IC50 values of S3I-301 treated MCF-7 and MDA-MB-468 cells cultured with/without adipocytes for 48 hours. B) Cell viability curves for the IC50 values of U0126 treated MCF-7 and MDA-MB-468 cells cultured with/without adipocytes for 48 hours. C. Representative western blot images of STAT3 and pSTAT3 in MCF-7 and MDA-MB-468 cell following treatment with the STAT3 inhibitor S3I-201. D. Representative western blot images of ERK1/2 and pERK in MCF-7 and MDA-MB-468 cell following treatment with the ERK1/2 inhibitor UO126.

Supplementary figure 5

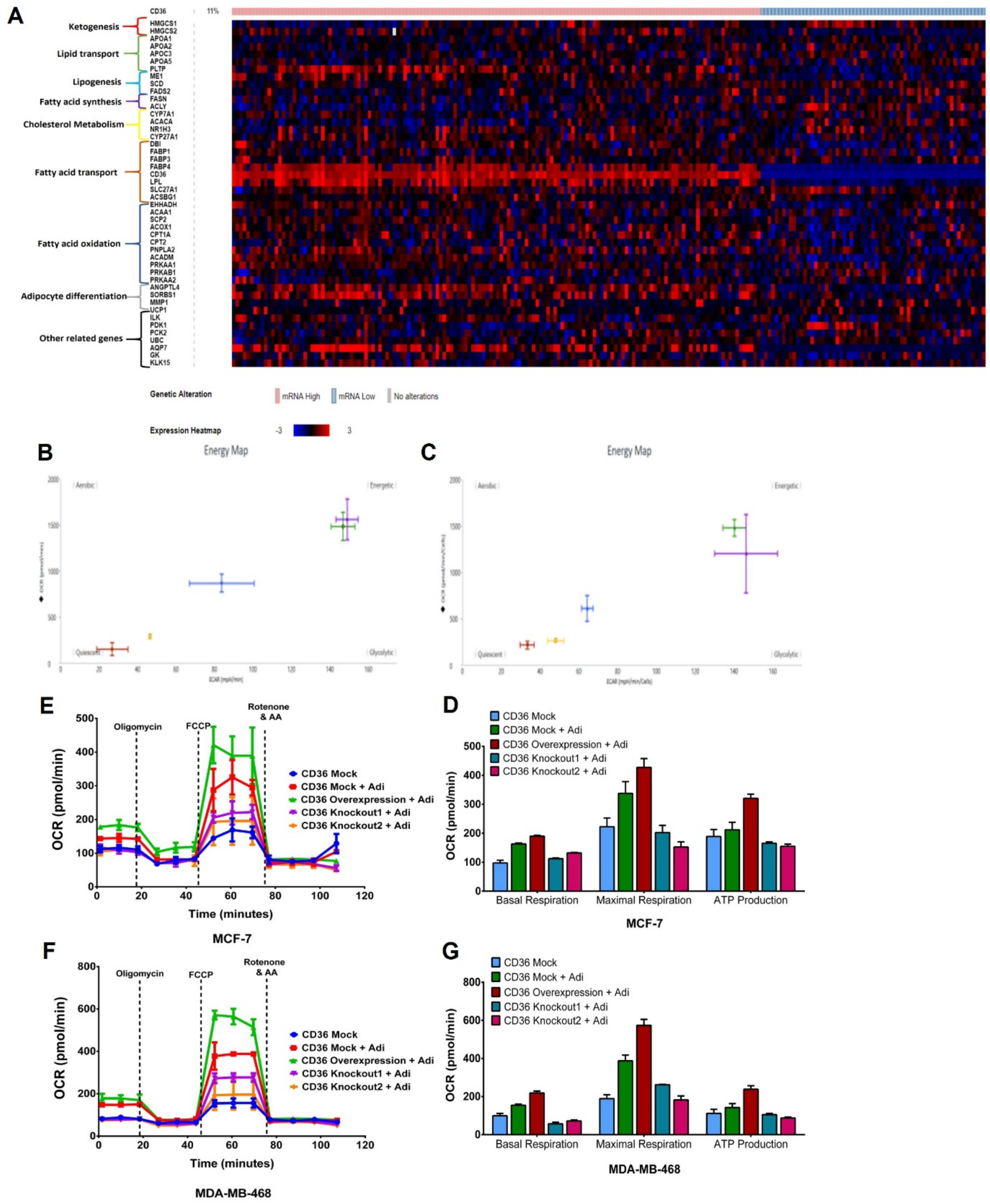

Supplementary figure 5: Upregulation of CD36 reprograms breasts cancer cell metabolism. A) Heatmap of key genes involved in ketogenesis, lipid transport, fatty acid synthesis, cholesterol metabolism, fatty acid transport, fatty acid oxidation, adipocyte differention and other related genes in a breast cancer cohort from the TCGA. B and C) Metabolic profile of MCF-7(B) and MDA-MB-468(C) cells with/without CD36 ablation cultured with/without adipocytes. Adipocytes induces shift in mitochondrial dynamics and energy production. Cellular phenotype plot comparing OCR on the y-axis

and ECAR on the x-axis. E and F) XFe24 Seahorse mitochondrial stress test on of MCF-7 (E) and MDA-MB-468 (F) cells with/without CD36 ablation cultured with/without adipocytes in real time under basal conditions and in response to mitochondrial inhibitors (O, oligomycin; F, FCCP; A, antimycin). D and G) Seashore measurement of basal respiration, maximal respiration, and ATP production rate in MCF-7 (D) and MDA-MB-468 (G) cells with/without CD36 ablation cultured with/without adipocytes.

Supplementary figure 6

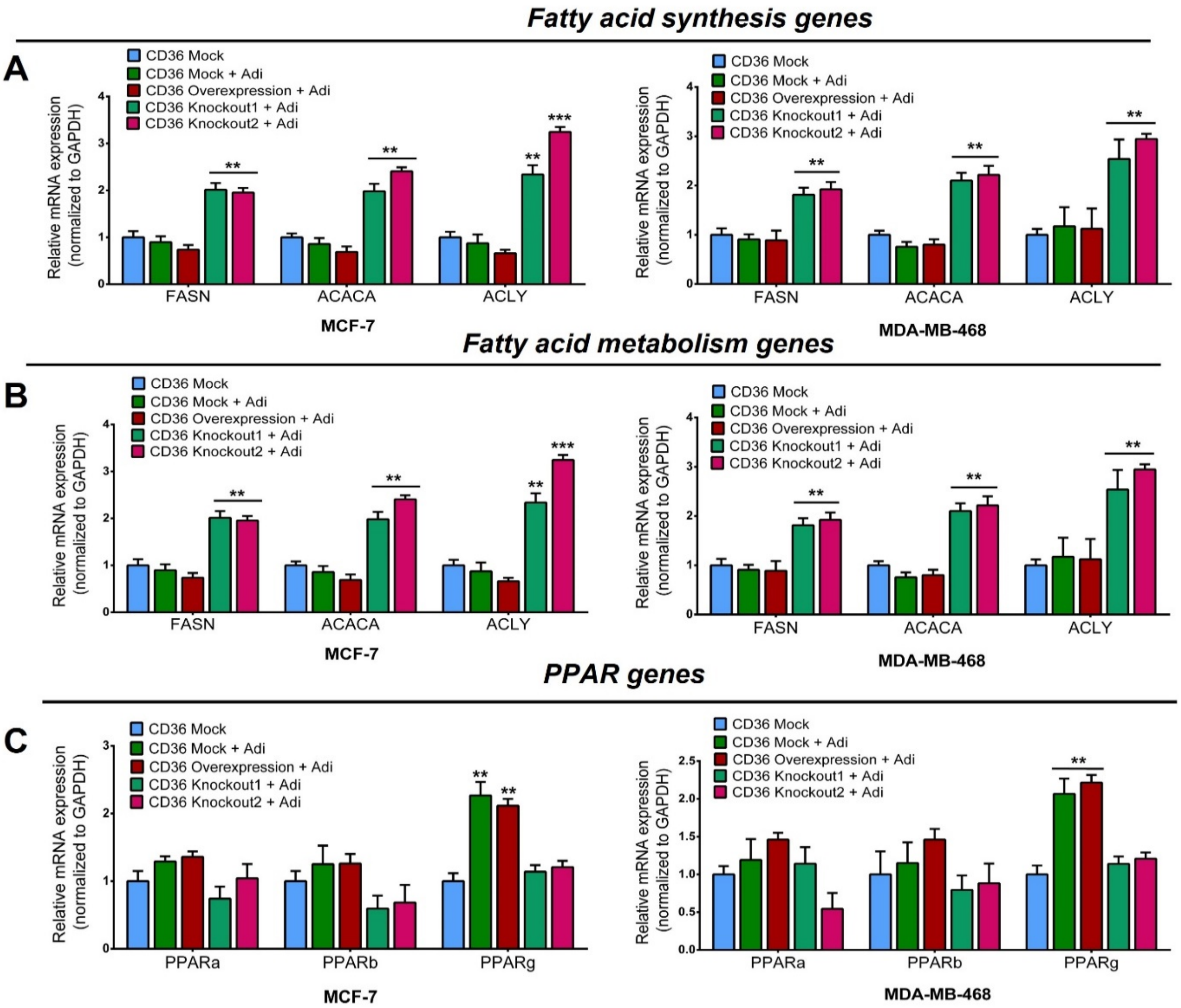

Supplementary figure 6: Quantitative expression of metabolic associated genes. A) Quantitative RT-PCR (qRT-PCR) comparing the expression of genes involved in lipid metabolism (CPT1a, ATGL and AMPK) in MCF-7 and MDA-MB-468 cells co-cultured with adipocytes with/without CD36 ablation. B) qRT-PCR comparing the expression of genes involved in lipid synthesis (FASN, ACACA and ACLY) in MCF-7 and MDA-MB-468 breast cancer cells co-cultured with adipocytes with/without CD36 ablation. C) qRT-PCR comparing the expression of PPARα, PPARβ and PPARγ in MCF-7 and MDA-MB-468 cells co-cultured with adipocytes with/without CD36 ablation. Relative mRNA

expressions were normalized to GADPH. All results are representative of 3 independent experiments. (qRT-PCR data represent mean ± SEM; ***p < 0,001; **p < 0.01; *p < 0.05).

Supplementary figure 7

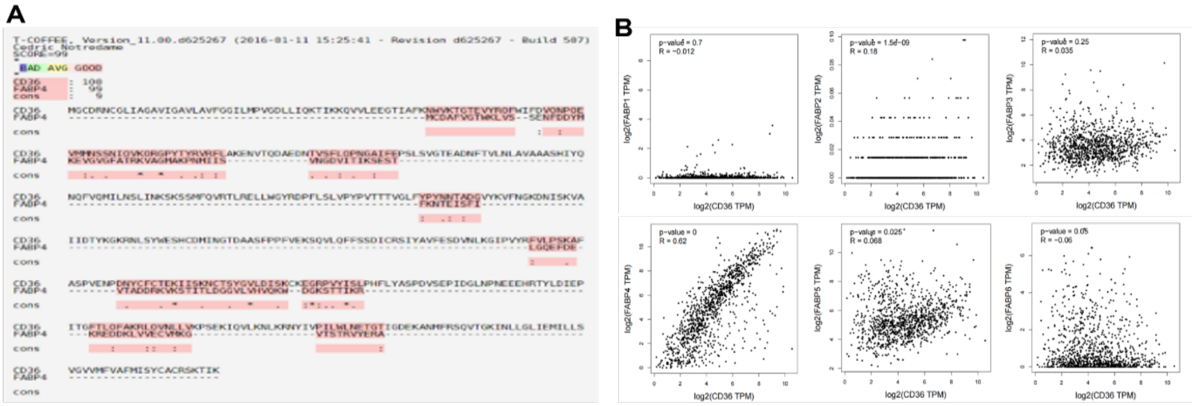

C

TCGA tumour	Gene ID	Pearson Correlation	TCGA tumour	Gene ID	Pearson Correlation	TCGA tumour	Gene ID	Pearson Correlation	TCGA tumour	Gene ID	Pearson Correlation
**BRCA (Breast Cancer)**	FABP4<br>ADIPOQ<br>ADH1A<br>ADH1B<br>AOC3<br>LEP<br>PCK1<br>LPL<br>RBP4<br>PDK4	0.77<br>0.74<br>0.72<br>0.71<br>0.71<br>0.70<br>0.64<br>0.64<br>0.63<br>0.61	**Pancreatic adenocarcinoma (PAAD)**	ADH1B<br>TEK<br>LOC339524<br>STARD8<br>FABP4<br>CELF2<br>PRKAR2B<br>ABCD2<br>LRRC33<br>RHOJ	0.75<br>0.75<br>0.74<br>0.73<br>0.73<br>0.72<br>0.72<br>0.71<br>0.71<br>0.70	**Cholangiocarcinoma (CHOL)**	FABP5<br>PLA2G4A<br>ACSL5<br>GPAT2<br>PTGS2<br>MTHFD2<br>NUS1<br>PLEKHG1<br>ST8SIA1<br>CD19	0.71<br>0.66<br>0.64<br>0.64<br>0.63<br>0.60<br>0.59<br>0.59<br>0.59<br>0.57	**Head and Neck squamous cell carcinoma (HNSC)**	LIPN<br>FABP4<br>PNPLA1<br>LIPK<br>THEM5<br>AADACL2<br>RDH12<br>PLA2G4F<br>LIPM<br>ACSL1	0.46<br>0.45<br>0.44<br>0.42<br>0.42<br>0.41<br>0.41<br>0.38<br>0.37<br>0.37
**ACC (Adrenocortical Carcinoma)**	ANGPTL1<br>SCARF1<br>SLC38A8<br>ASAH1<br>FABP4<br>ACAT1<br>ALDH3B1<br>HPGDS<br>PEX7<br>LEPROTL1	0.51<br>0.50<br>0.49<br>0.48<br>0.43<br>0.43<br>0.43<br>0.41<br>0.41<br>0.41	**Stomach and Esophageal Carcinoma (STES)**	FABP4<br>LOC339524<br>ADH1B<br>TEK<br>GPIHBP1<br>ADIPOQ<br>AOC3<br>RHOJ<br>THSD7A<br>ENPP2	0.62<br>0.57<br>0.53<br>0.52<br>0.52<br>0.51<br>0.51<br>0.50<br>0.50<br>0.50	**Colon adenocarcinoma (COAD)**	C3AR1<br>LOC339524<br>ST8SIA4<br>ADIPOQ<br>SRGN<br>ENPP2<br>FABP4<br>TEK<br>CYP1B1<br>PIK3R5	0.71<br>0.71<br>0.69<br>0.68<br>0.68<br>0.67<br>0.67<br>0.67<br>0.66<br>0.66	**Liver hepatocellular carcinoma (LIHC)**	ACSL5<br>ABHD1<br>FABP4<br>IRS1<br>ALDH1A1<br>AMACR<br>ABHD6<br>PTGR1<br>EHHADH<br>HAO1	0.54<br>0.50<br>0.47<br>0.46<br>0.45<br>0.45<br>0.45<br>0.43<br>0.42<br>0.41
**Cervical squamous cell carcinoma and endocervical adenocarcinoma (CESC)**	RDH12<br>FABP4<br>NOD2<br>FABP5<br>SLC39A2<br>PNLIPRP3<br>LRP1<br>SLC37A2<br>GM2A<br>FABP5L3	0.52<br>0.51<br>0.49<br>0.45<br>0.44<br>0.44<br>0.44<br>0.42<br>0.42<br>0.40	**Stomach adenocarcinoma (STAD)**	TEK<br>FABP4<br>ADH1B<br>LOC339524<br>STARD8<br>ENPP2<br>RHOJ<br>THSD7A<br>GPIHBP1<br>AOC3	0.70<br>0.69<br>0.65<br>0.65<br>0.64<br>0.63<br>0.63<br>0.62<br>0.61<br>0.59	**Colonrectal adenocarcinoma (COADREAD)**	C3AR1<br>LOC339524<br>ST8SIA4<br>SRGN<br>ENPP2<br>ADIPOQ<br>FABP4<br>TEK<br>PLEK<br>CYP1B1	0.70<br>0.69<br>0.69<br>0.67<br>0.67<br>0.67<br>0.65<br>0.65<br>0.64<br>0.64	**Lung squamous cell carcinoma (LUSC)**	SLC39A8<br>GKN2<br>AGTR2<br>TEK<br>PLA2G1B<br>FABP4<br>PDK4<br>RHOJ<br>NPC2<br>LPL	0.52<br>0.51<br>0.48<br>0.47<br>0.46<br>0.46<br>0.46<br>0.45<br>0.45<br>0.45
**Ovarian serous cystadenocarcinoma (OV)**	FABP4<br>AOC3<br>ADIPOQ<br>ADH1B<br>THBS2<br>STARD8<br>AGTR1<br>ANGPTL2<br>PDGFRB<br>THBD	0.72<br>0.66<br>0.64<br>0.60<br>0.56<br>0.53<br>0.53<br>0.52<br>0.51<br>0.50	**Thyroid carcinoma (THCA)**	FABP4<br>AGTR1<br>CETP<br>FLT1<br>PAMR1<br>ADIPOR2<br>STARD8<br>EXOC3L2<br>GPIHBP1<br>PIK3R3	0.80<br>0.76<br>0.70<br>0.67<br>0.65<br>0.64<br>0.63<br>0.63<br>0.60<br>0.59	**Esophageal carcinoma (ESCA)**	FABP4<br>THBD<br>ELOVL4<br>LIPK<br>LEPR<br>SOCS3<br>ST8SIA1<br>CES1<br>TBCEL<br>SLC37A2	0.53<br>0.48<br>0.43<br>0.41<br>0.41<br>0.40<br>0.40<br>0.39<br>0.39<br>0.39	**Lung adenocarcinoma (LUAD)**	TEK<br>STARD8<br>GPIHBP1<br>FABP4<br>RHOJ<br>ADH1B<br>LIPA<br>CLEC3B<br>THSD1<br>AOC3	0.57<br>0.55<br>0.55<br>0.54<br>0.50<br>0.50<br>0.50<br>0.49<br>0.48<br>0.48

D

Gene	Function	Tumour types	Highest PC	Lowest PC
**Fatty acid Binding protein 4** (FABP4)	Family of small, highly conserved, cytoplasmic proteins that bind long-chain fatty acids and other hydrophobic ligands. FABPs roles include fatty acid uptake, transport, and metabolism.	15	0.80 THCA	0.43 ACC
**TEK Receptor Tyrosine Kinase** (TEK)	Tyrosine-protein kinase that acts as cell-surface receptor for ANGPT1, ANGPT2 and ANGPT4 and regulates angiogenesis, endothelial cell survival, proliferation, migration, adhesion and cell spreading, reorganization of the actin cytoskeleton, maintenance of vascular quiescence.	7	0.75 PAAD	0.47 LUSC
**Adiponectin, C1Q And Collagen Domain Containing** (ADIPOQ)	Adipokine involved in the control of fat metabolism and insulin sensitivity, anti-atherogenic and anti-inflammatory activities. Stimulates AMPK phosphorylation and activation in the liver and the skeletal muscle, enhancing glucose utilization and fatty-acid combustion.	6	0.74 BRCA	0.38 BLCA
**Alcohol Dehydrogenase 1B** (ADH1B)	This enzyme family metabolize a wide variety of substrates, including ethanol, retinol, other aliphatic alcohols, hydroxysteroids, and lipid peroxidation products.	6	0.75 PAAD	0.30 BLCA
**LOC339524**	This locus has a high frequency of deletions, amplifications, and translocations, which are associated with tumours.	6	0.74 PAAD	0.30 BLCA
**StAR Related Lipid Transfer Domain Containing 8** (STARD8)	Encodes a member of a subfamily of Rho GTPase activating proteins that contain a steroidogenic acute regulatory protein related lipid transfer domain. The encoded protein localizes to focal adhesions and may be involved in regulating cell morphology. This protein may also function as a tumor suppressor.	6	0.74 PAAD	0.31 BLCA
***AOC3* Gene(Protein Coding) Amine Oxidase Copper Containing 3** (AOC3	Copper amine oxidases catalyze the oxidative conversion of amines to aldehydes in the presence of copper and quinone cofactor. The encoded protein is localized to the cell surface, has adhesive properties as well as monoamine oxidase activity, and may be involved in leukocyte trafficking.	5	0.66 OV	0.48 LUAD
**Ras Homolog Family Member J** (RHOJ)	Plasma membrane-associated small GTPase specifically involved in angiogenesis. Required for endothelial cell migration during vascular development.	5	0.70 PAAD	0.45
**Lipoprotein Lipase** (LPL)	Key enzyme in triglyceride metabolism. Catalyzes the hydrolysis of triglycerides from circulating chylomicrons and very low density lipoproteins (VLDL), and thereby plays an important role in lipid clearance from the blood stream, lipid utilization and storage.	3	0.64 BRCA	0.33 BLCA

Supplementary figure 7: CD36 directly interacts with FABP4. A) Amino acid ssequence alignment of CD36 and FABP4 by T-coffee, shows several potential interacting motifs between CD36 and FABP4.

B) Correlation Matrix for the correlation between CD36 and various FABP isoforms in the TCGA database. FABP4 presents the highest positive correlation with CD36. Correlation plots were generated in the GEPIA online portal. C) Pearson correlation coefficient for top 10 genes correlating to CD36 expression in 16 cancer cohort from TCGA. D) Pearson correlation coefficient for genes commonly correlating with increased CD36 expression in 16 cancer cohorts from the TCGA database.

Supplementary figure 8

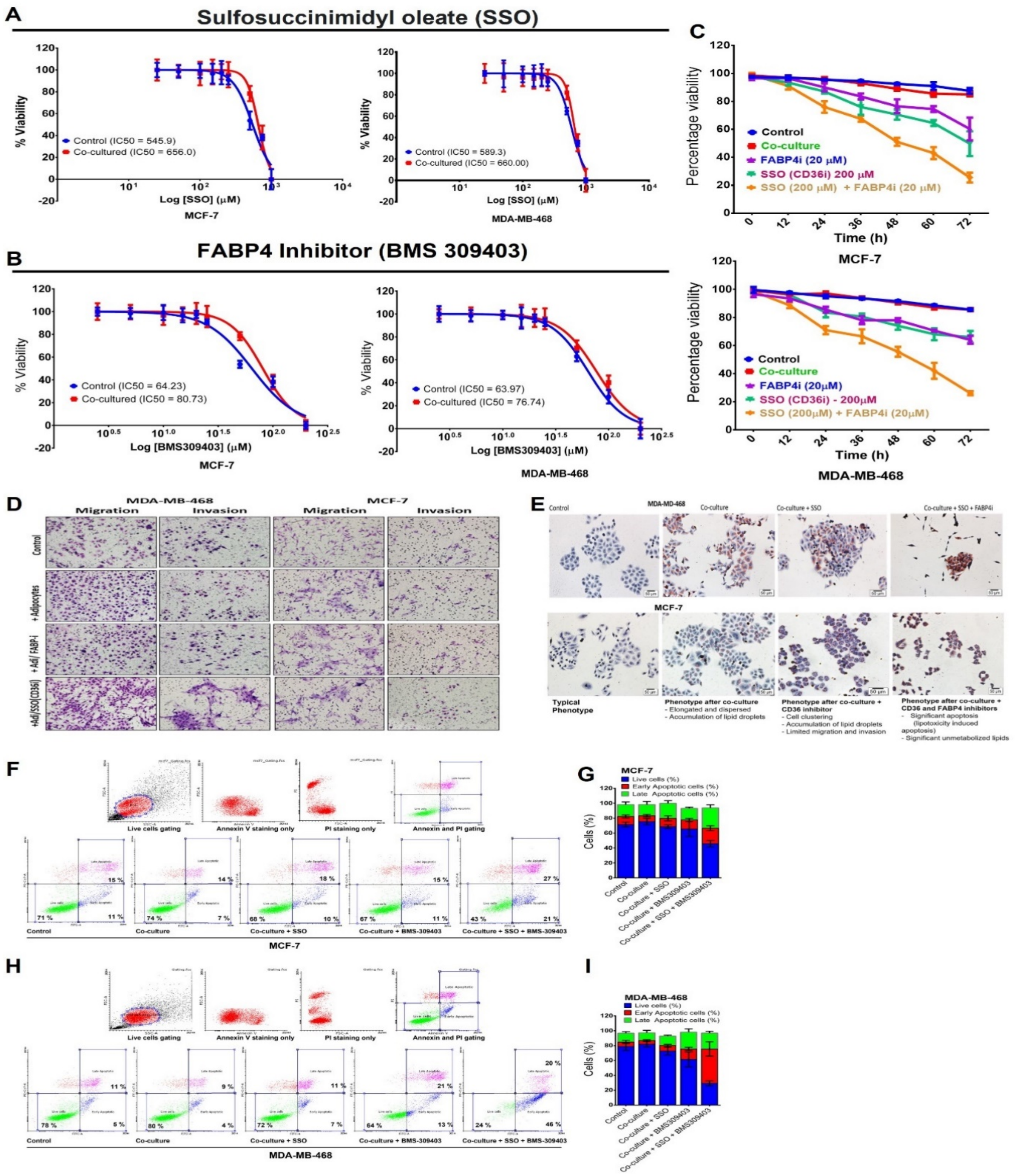

Supplementary figure 8: Combined inhibition of CD36 and FABP4 induces apoptosis in breast cancer cells. A) IC50 estimation for sulfosuccinimidyl oleate (SSO) treated MCF-7 and MDA-MB-468

cells cultured with/without adipocyte conditioned media for 48 hours. B) IC50 estimation for the FABP4 inhibitor (BMS 309403) treated MCF-7 and MDA-MB-468 cells cultured with/without adipocyte conditioned media for 48 hours. C) Cell viability curves for effect of SSO and BMS-309403 on breast cancer cells (MDA-MB-468 and MCF-7) following 6, 12, 24, 36, 48 and 72-hour exposure. D) Representative images of MCF-7 and MDA-MB-468 migration and invasion (x200 magnification) after co-culture with adipocytes. E) Representative images for oil red O staining of MCF-7 and MDA-MB-468 cells following treatment with SSO and BMS-309403. F and H) Representative images of Annexin V/PI apoptosis assay in MCF-7 (F) and MDA-MB-468 (H) cells treated with SSO and BMS-309403. G and I) Quantitative estimation of cell counts from Annexin V/PI apoptosis assay in MCF-7 (G) and MDA-MB-468 (I) cells treated with SSO and BMS-309403.

Supplementary figure 9

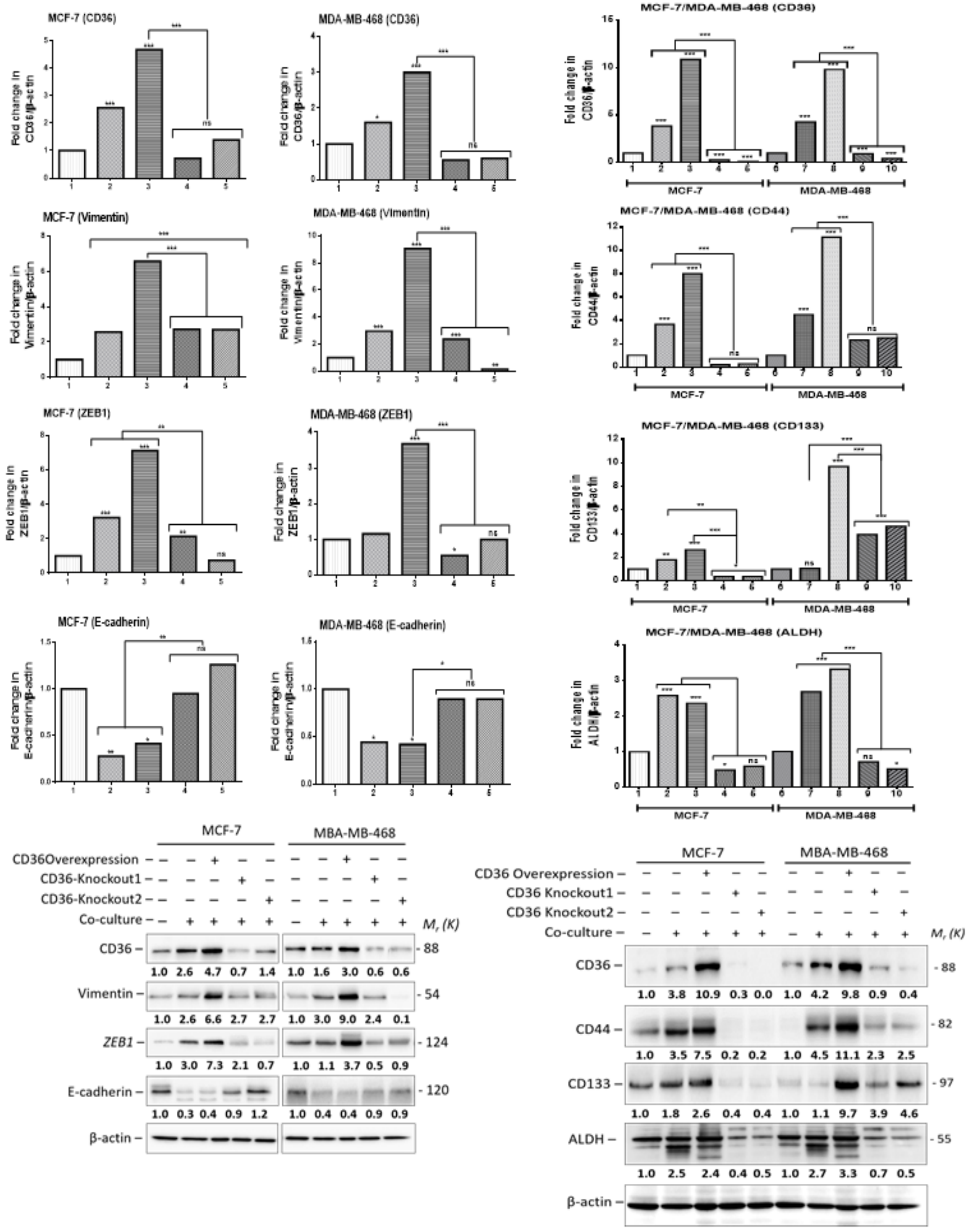

Supplementary figure 9: Quantification of western blot images for Figure 2B and Figure 2F.

Supplementary figure 10:

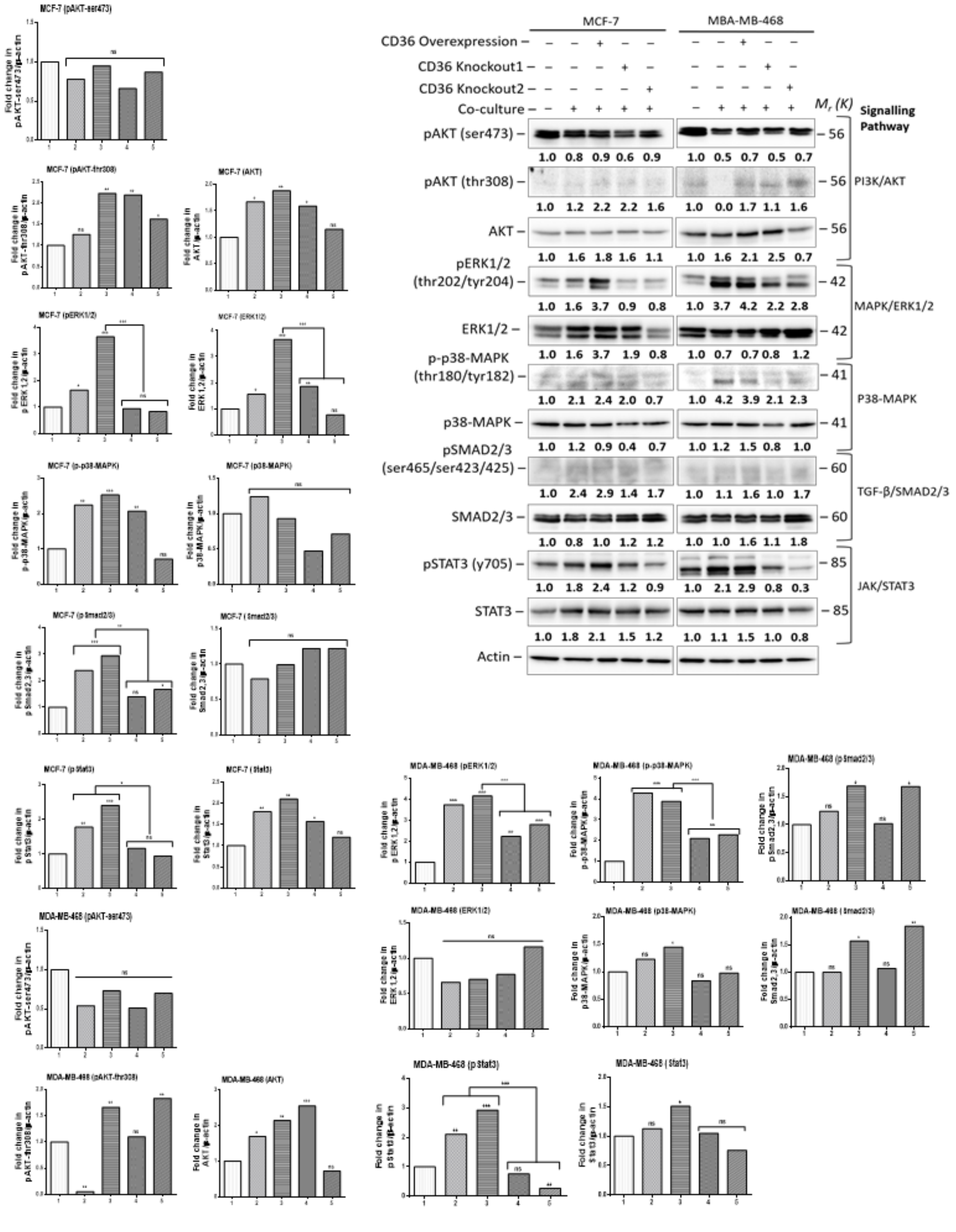

Supplementary figure 10: Quantification of western blot images for Figure 4A.

Supplementary figure 11:

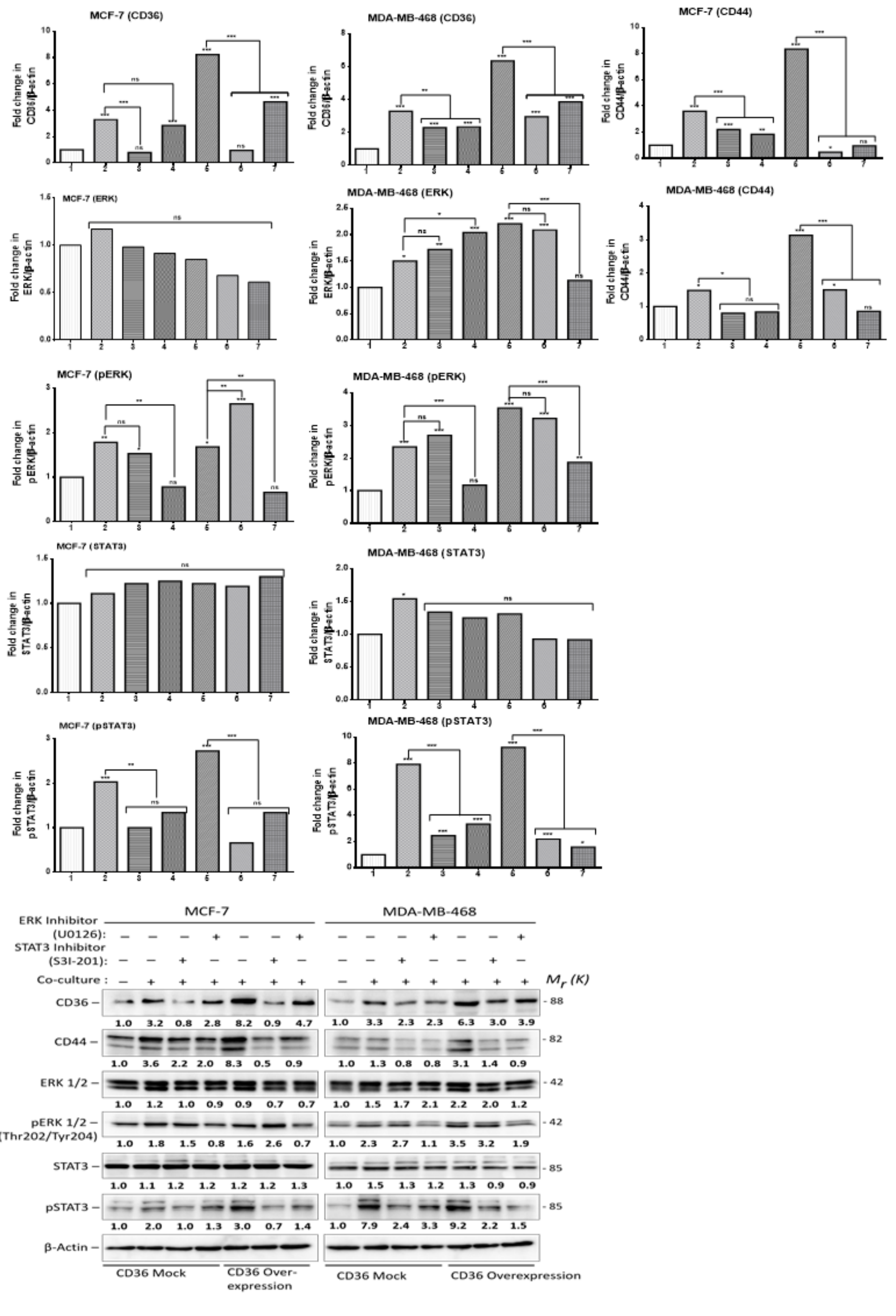

Supplementary figure 11: Quantification of western blot images for Figure 4B.

Supplementary figure 12:

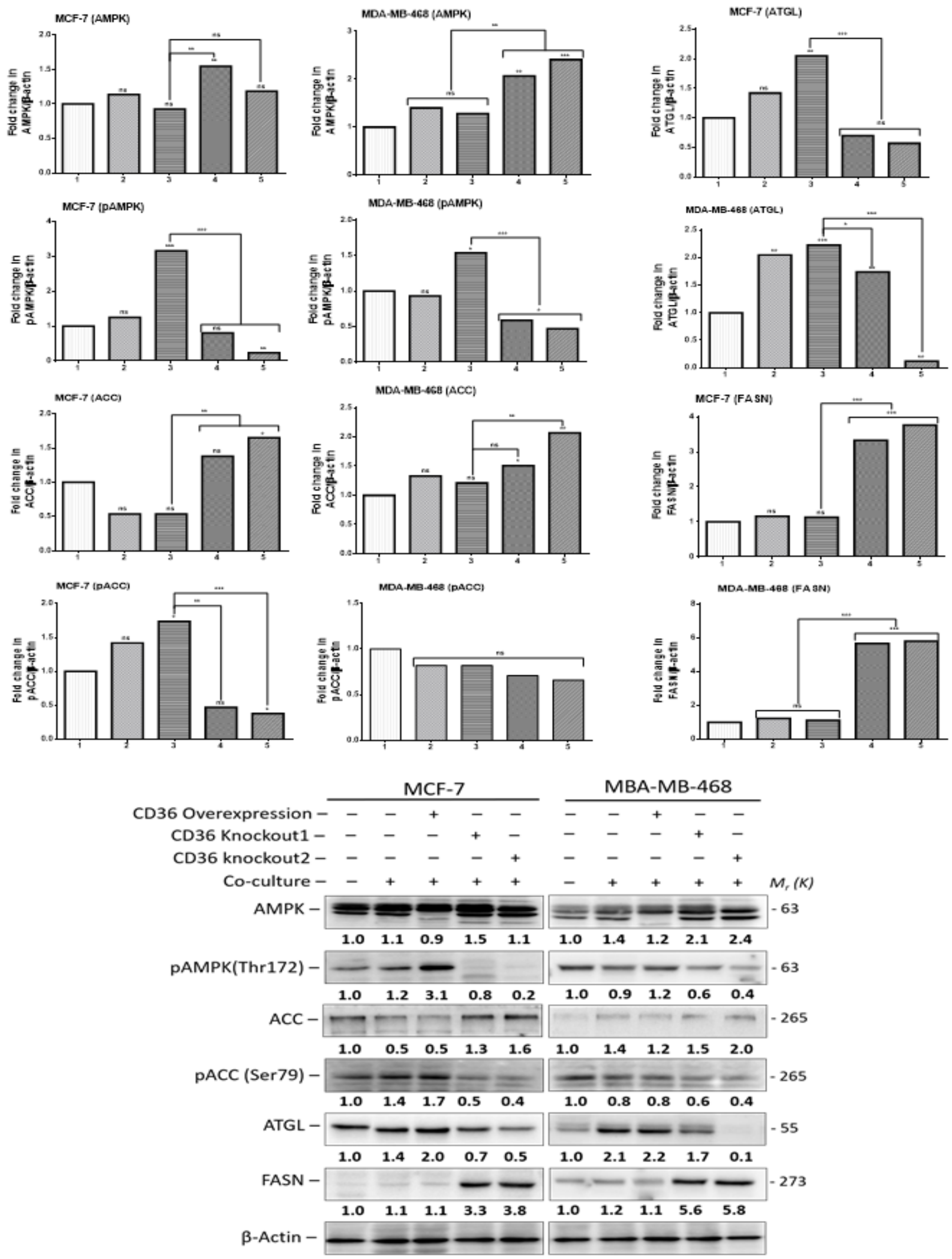

Supplementary figure 12: Quantification of western blot images for Figure 6E.

Supplementary figure 13:

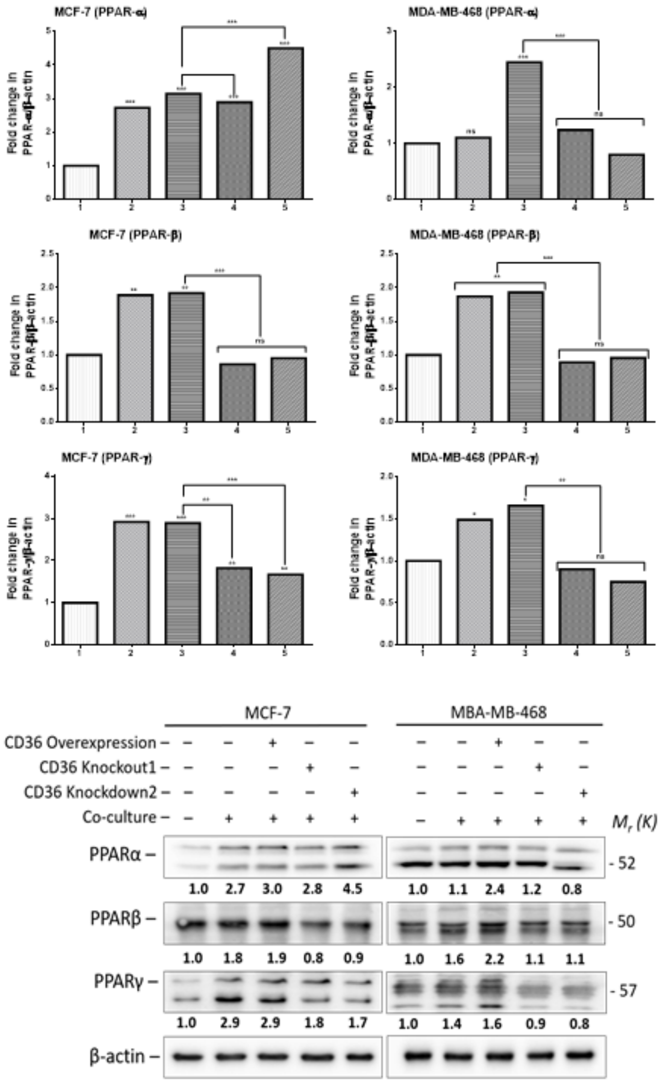

Supplementary figure 13: Quantification of western blot images for Figure 6F.

Supplementary figure 14:

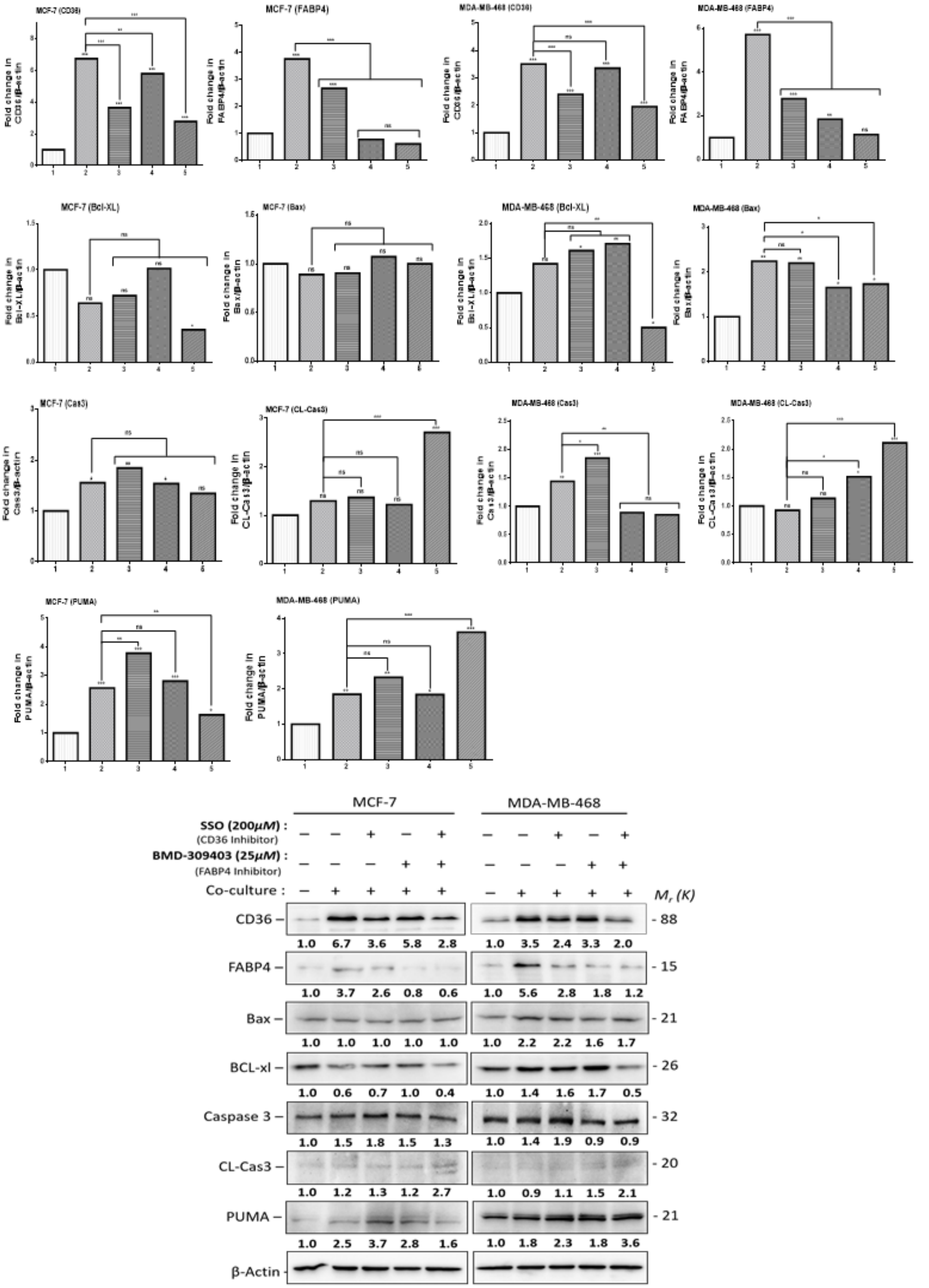

Supplementary figure 14: Quantification of western blot images for Figure 8E.

Supplementary figure 15: Uncropped western blot Images

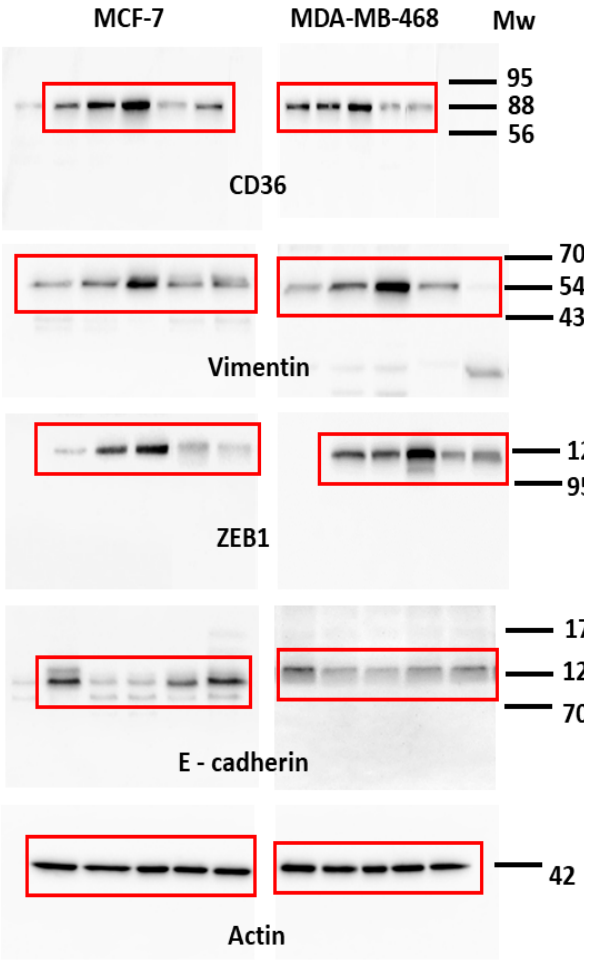

Figure 2F full blots

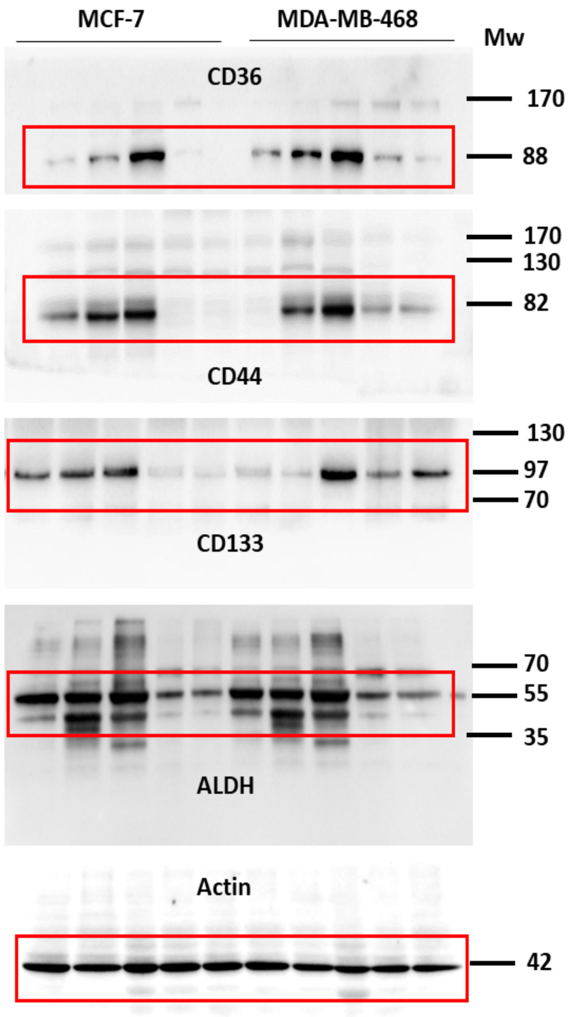

Figure 4A full blots

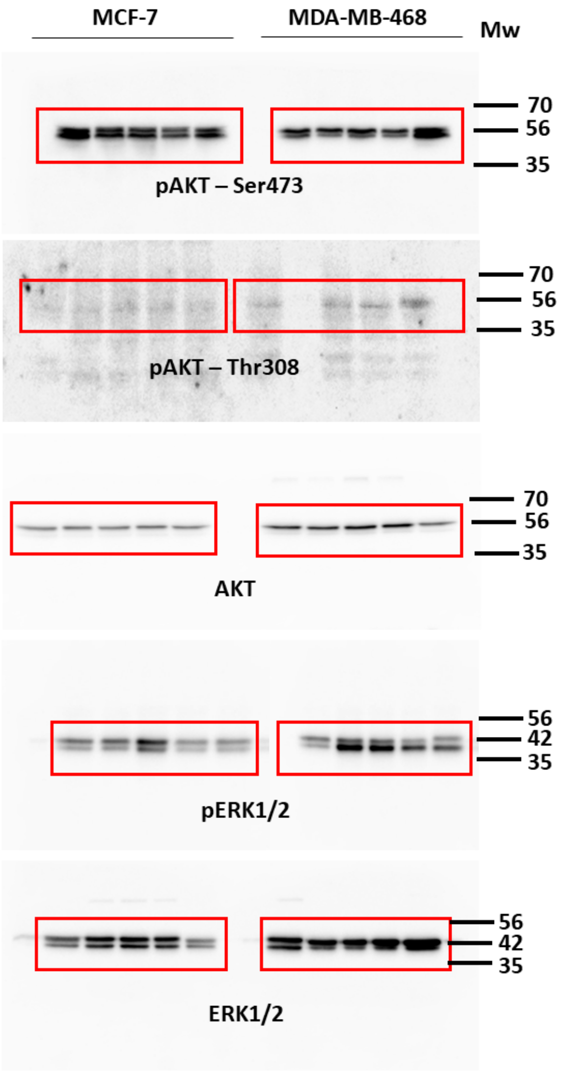

Figure 4A full blots continued

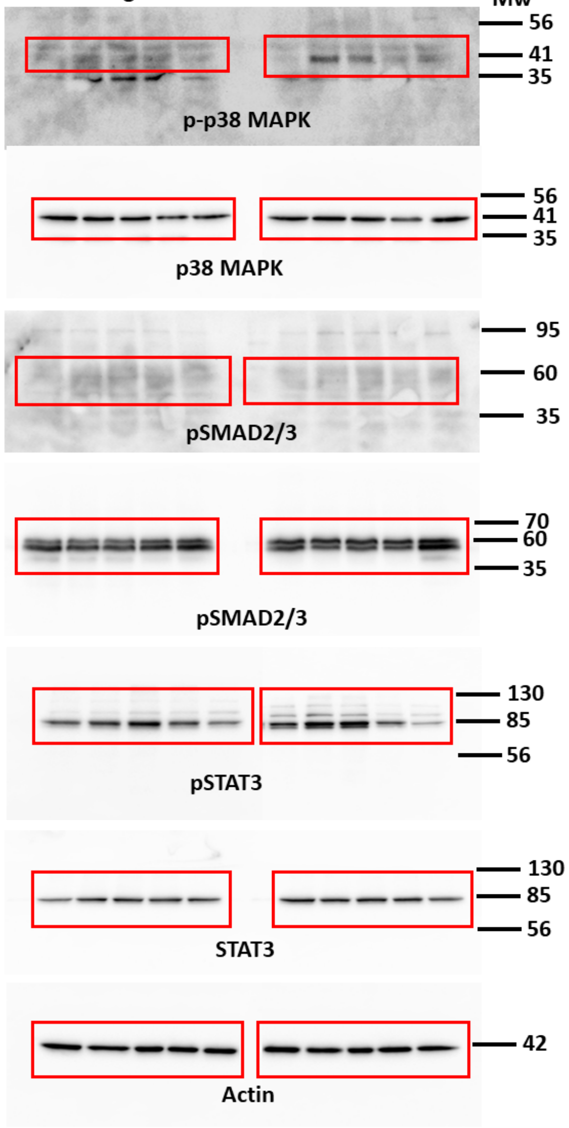

Figure 4B full blots

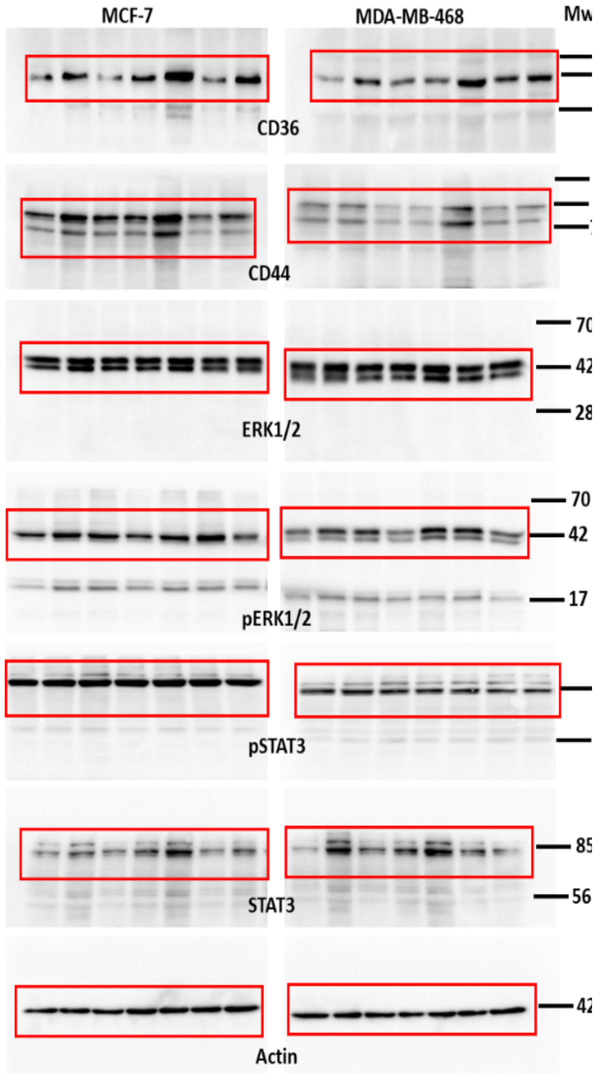

Figure 4C full blots

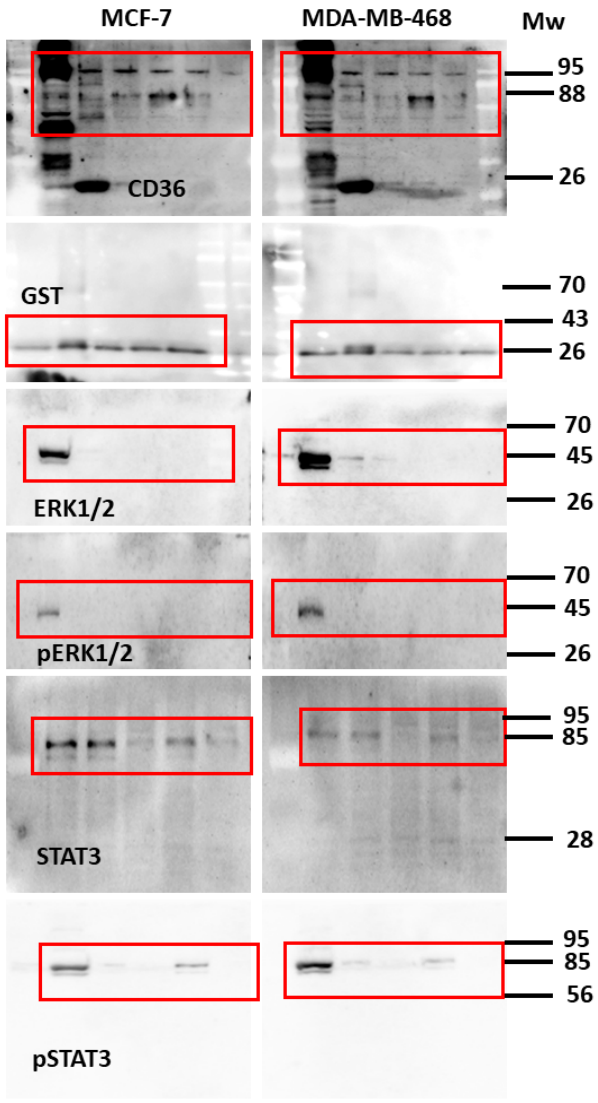

Figure 5E full blots

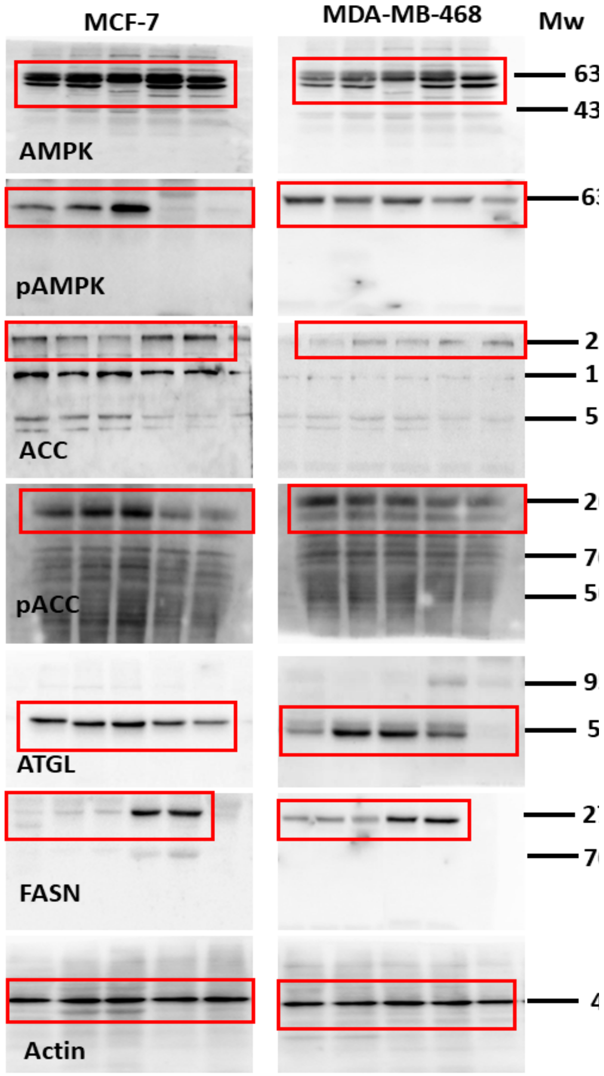

Figure 5F full blots

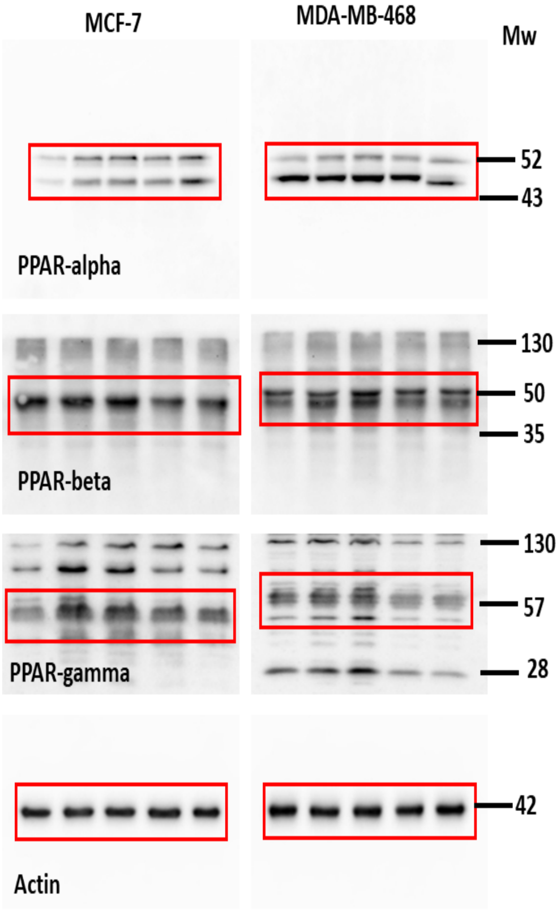

Figure 6B full blots

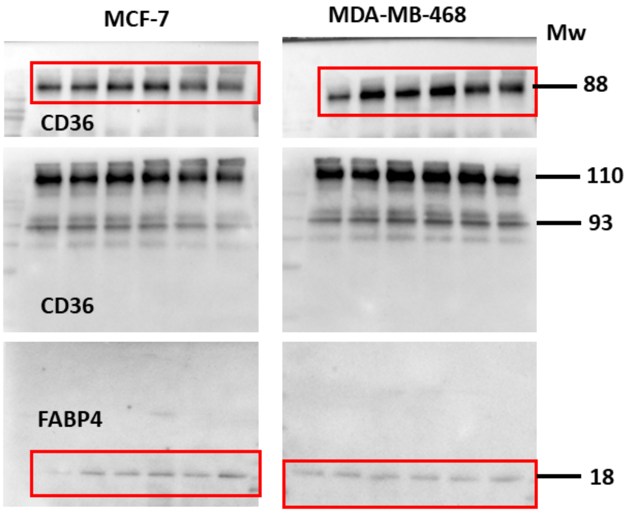

Figure 6C (ii) full blots

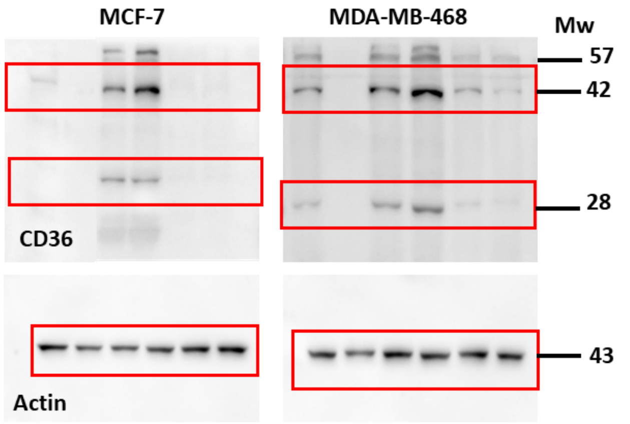

Figure 6D full blots

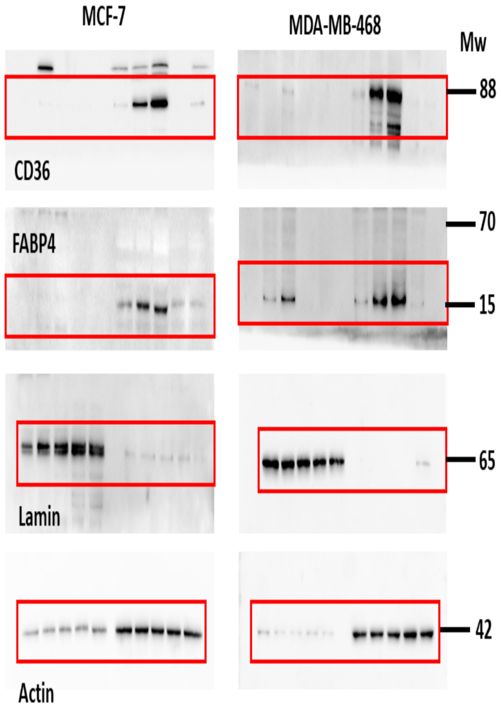

Figure 7F full blots

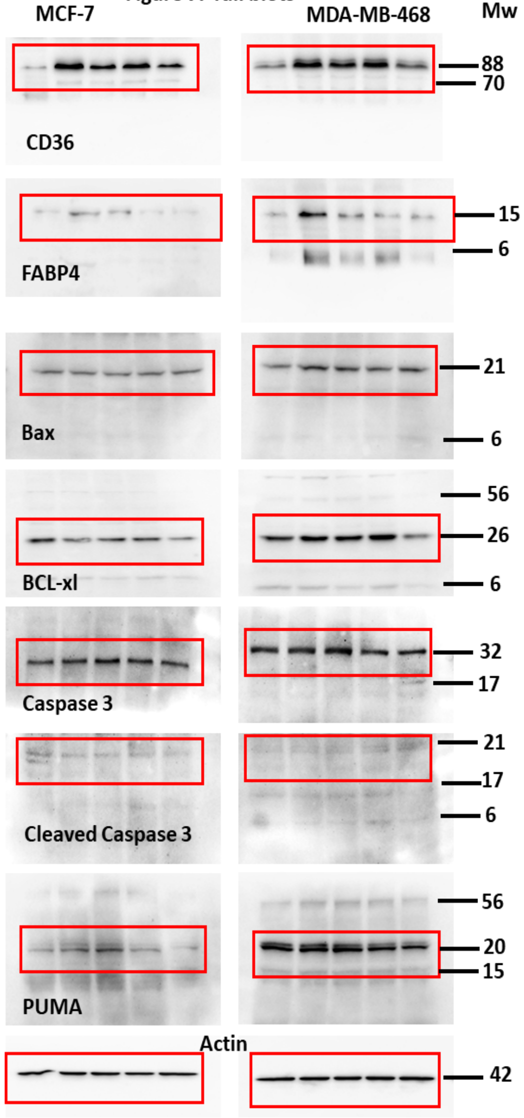

Supplementary figure 3C and 3D full blots

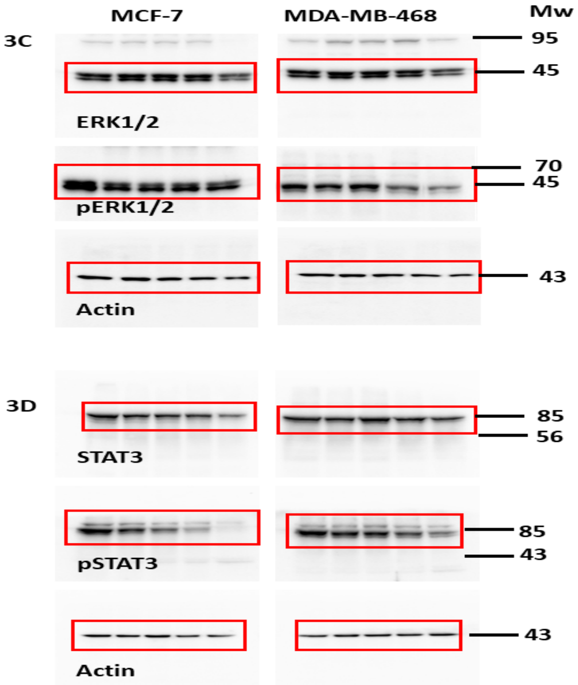

Supplementary figure 1M full blots

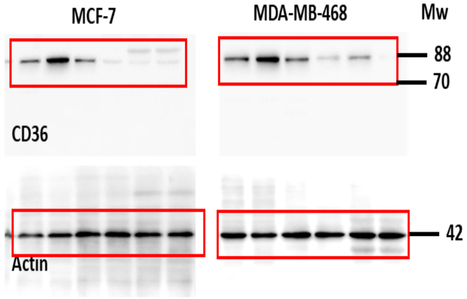

Supplement: Supplementary file 1 — Supplementary Information [file 41523_2021_324_MOESM1_ESM.pdf]
