# Supplementary material for: Interaction between CD36 and FABP4 modulates adipocyte-induced fatty acid import and metabolism in breast cancer
Source: NPJ Breast Cancer. 2021 Sep 24;7:129. doi: 10.1038/s41523-021-00324-7 (PMC8463699; doi:10.1038/s41523-021-00324-7)
Supplement: Supplementary file 2 — Reporting Summary [file 41523_2021_324_MOESM2_ESM.pdf]

nature research

Corresponding author(s): Junjeong Choi

Last updated by author(s): Aug 4, 2021

Reporting Summary

Nature Research wishes to improve the reproducibility of the work that we publish. This form provides structure for consistency and transparency in reporting. For further information on Nature Research policies, see Authors & Referees and the Editorial Policy Checklist.

Statistics

For all statistical analyses, confirm that the following items are present in the figure legend, table legend, main text, or Methods section.

n/a	Confirmed	
☐	☒	The exact sample size (*n*) for each experimental group/condition, given as a discrete number and unit of measurement
☒	☐	A statement on whether measurements were taken from distinct samples or whether the same sample was measured repeatedly
☐	☒	The statistical test(s) used AND whether they are one- or two-sided<br>*Only common tests should be described solely by name; describe more complex techniques in the Methods section.*
☒	☐	A description of all covariates tested
☐	☒	A description of any assumptions or corrections, such as tests of normality and adjustment for multiple comparisons
☐	☒	A full description of the statistical parameters including central tendency (e.g. means) or other basic estimates (e.g. regression coefficient) AND variation (e.g. standard deviation) or associated estimates of uncertainty (e.g. confidence intervals)
☒	☐	For null hypothesis testing, the test statistic (e.g. *F*, *t*, *r*) with confidence intervals, effect sizes, degrees of freedom and *P* value noted<br>*Give P values as exact values whenever suitable.*
☒	☐	For Bayesian analysis, information on the choice of priors and Markov chain Monte Carlo settings
☒	☐	For hierarchical and complex designs, identification of the appropriate level for tests and full reporting of outcomes
☒	☐	Estimates of effect sizes (e.g. Cohen's *d*, Pearson's *r*), indicating how they were calculated

Our web collection on statistics for biologists contains articles on many of the points above.

Software and code

Policy information about availability of computer code

Data collection	The Cancer Genome Atlas Database, rVista (http://rvista.dcode.org), CONTRA v2 (http://www.dmbr.ugent.be/prx/bioit2-public/contrav2/index.php), NCBI sequence finder tool, Morpheus online tool (https://software.broadinstitute.org/morpheus/), The molecular signature database (MsigDB) website (http://software.broadinstitute.org/gsea/msigdb/genesets.jsp?collection=C5).
Data analysis	GraphPad Prism 6.0 (GraphPad Software Inc.)

For manuscripts utilizing custom algorithms or software that are central to the research but not yet described in published literature, software must be made available to editors/reviewers. We strongly encourage code deposition in a community repository (e.g. GitHub). See the Nature Research guidelines for submitting code & software for further information.

Data

Policy information about availability of data

All manuscripts must include a data availability statement. This statement should provide the following information, where applicable:

- Accession codes, unique identifiers, or web links for publicly available datasets
- A list of figures that have associated raw data
- A description of any restrictions on data availability

The uncropped blots presented in this study are available in Supplementary Figure 7. All additional data is available from the corresponding author upon reasonable request.

Field-specific reporting

Please select the one below that is the best fit for your research. If you are not sure, read the appropriate sections before making your selection.

☒ Life sciences ☐ Behavioural & social sciences ☐ Ecological, evolutionary & environmental sciences

For a reference copy of the document with all sections, see nature.com/documents/nr-reporting-summary-flat.pdf

Life sciences study design

All studies must disclose on these points even when the disclosure is negative.

Sample size	The in vitro cell culture experiments, we used 2-3 biological samples in each experiment.
Data exclusions	No data were excluded.
Replication	All attempts at replication were successful.
Randomization	N/A
Blinding	Investigators were blinded to grade tumour staining

Reporting for specific materials, systems and methods

We require information from authors about some types of materials, experimental systems and methods used in many studies. Here, indicate whether each material, system or method listed is relevant to your study. If you are not sure if a list item applies to your research, read the appropriate section before selecting a response.

Materials & experimental systems

n/a	Involved in the study	
☐	☒	Antibodies
☐	☒	Eukaryotic cell lines
☒	☐	Palaeontology
☐	☒	Animals and other organisms
☒	☐	Human research participants
☐	☒	Clinical data

Methods

n/a	Involved in the study	
☒	☐	ChIP-seq
☐	☒	Flow cytometry
☒	☐	MRI-based neuroimaging

Antibodies

Antibodies used	CD36 - Proteintech (Cat. no. 18836-1-AP), Vimentin - Proteintech (Cat. no. 60330-1-Ig), ZEB1 - Sigma-Aldrich (Cat. no. HPA027524), E-cadherin - Cell Signalling (cat. no. 3195), CD44 - Cell Signalling (Cat. no. 3570), CD133- Abcam (Cat. no. Ab19898), ALDH - BD (Cat. no. 611195), AKT- Cell Signalling (cat. no. 9272), pAKT (ser473) - Cell Signalling (Cat. no. 9271), pAKT (Thr308) - cell Signalling (cat. no. 5106), ERK1/2 - Cell Signalling (cat. no. 9102), pERK1/2 (Thr202/Tyr204) - Cell Signalling (cat. no. 9191), P38-MAPK - Santa cruz (cat. no. Sc-728), pP38-MAPK - Cell Signalling (Cat. no. 9211), SMAD 2/3 - Cell Signalling (cat. no. 3102), pSMAD2/3 - Cell Signalling (Cat. no. 8828), STAT3 - Cell Signalling (Cat. no. 9139), pSTAT3 (Y705) - Abcam (Cat. no. Ab76315), AMPK - ThermoFisher (Cat. no. MA5-15815), pAMPK (Thr172) - ThermoFisher (Cat. no. 44-1150G), ACC - Abcam (Cat. no. Ab70246), pACC (Ser79) - Cell Signalling (Cat. no. 11818), ATGL - Abcam (Cat. no. Ab207799), FASN - Abcam (Cat. no. Ab128856), PPAR-a - Santa Cruz (Cat. no. Sc-398394), PPAR-b - Santa Cruz (Cat. no. Sc-74440), PPAR-g - Cell Signalling (Cat. no. 2435), FABP4 - Santa Cruz (Cat. no. Sc-271529), BAX - Cell Signalling (Cat. no. 2772), BCL-xL - Cell Signalling BCL-xL Cell Signalling (Cat. no. 2762), Caspase 3 - Cell Signalling (Cat. no. 9662), CL-Cas3 - Cell Signalling (Cat. no. 9661), PUMA - Santa Cruz (Cat. no. sc-374223), GST - Abcam (Cat. no. Ab19256), AlexaFluor 647 - abcam (Cat. no. Ab150083), Alexafluor 488 - Abcam (Cat. no. Ab150077), IgG (Mouse) - Santa cruz (Cat. no. Sc-2005), IgG (Rabbit) - Santa cruz (Cat. no. Sc-2004), a-lamin - Santa Cruz (Cat. no. Sc-518013), Actin - Santa Cruz (Cat. no. Sc-47778).
Validation	Each antibody was validated by data sheet from the manufacturer, literature's citations and previously published articles from the same group.

Eukaryotic cell lines

Policy information about cell lines

Cell line source(s)	Cell lines were purchased from ATCC.

Authentication	ATCC authenticated, and observation of morphology.
Mycoplasma contamination	Cell lines were negative for mycoplasma contamination.
Commonly misidentified lines (See ICLAC register)	No cell lines listed by ICLAC were used.

Animals and other organisms

Policy information about studies involving animals; ARRIVE guidelines recommended for reporting animal research

Laboratory animals	The study did not involve laboratory animals
Wild animals	The study did not involve wild animals.
Field-collected samples	The study did not involve samples collected from the field.
Ethics oversight	N/A

Note that full information on the approval of the study protocol must also be provided in the manuscript.

Clinical data

Policy information about clinical studies

All manuscripts should comply with the ICMJE guidelines for publication of clinical research and a completed CONSORT checklist must be included with all submissions.

Clinical trial registration	*Provide the trial registration number from ClinicalTrials.gov or an equivalent agency.*
Study protocol	*Note where the full trial protocol can be accessed OR if not available, explain why.*
Data collection	*Describe the settings and locales of data collection, noting the time periods of recruitment and data collection.*
Outcomes	*Describe how you pre-defined primary and secondary outcome measures and how you assessed these measures.*

Flow Cytometry

Plots

Confirm that:

- [x] The axis labels state the marker and fluorochrome used (e.g. CD4-FITC).
- [x] The axis scales are clearly visible. Include numbers along axes only for bottom left plot of group (a 'group' is an analysis of identical markers).
- [x] All plots are contour plots with outliers or pseudocolor plots.
- [ ] A numerical value for number of cells or percentage (with statistics) is provided.

Methodology

Sample preparation	To determine the proportion of CD36/CD44 expressing cells, cells co-cultured and control cells were harvested and resuspended to approximately 1-5x106 cells /ml in ice cold PBS. Cells are fixed with 4% formaldehyde for 15 mins at room temperature. Cells are resuspended in 100ul BSA/PBS and incubated with primary antibodies against CD36 and CD44 and for 1hr at room temperature. Cells are rinsed 3X and resuspended in ice cold PBS. Cells are incubated with Alexa Fluor 488 and Alexa Fluor 647-conjugated secondary antibody for another 30 minutes, rinsed and analysed.
Instrument	BD FACSAria™ III (BD Biosciences, San Jose, USA)
Software	Flowing software 2
Cell population abundance	Followed the manufacturer’s instruction
Gating strategy	Gating boundaries were designed equally in all of analyses.

- [x] Tick this box to confirm that a figure exemplifying the gating strategy is provided in the Supplementary Information.
